# Limited waterpower contributed to rise of steam power in British “Cottonopolis”

**DOI:** 10.1093/pnasnexus/pgae251

**Published:** 2024-07-16

**Authors:** Tara N Jonell, Peter Jones, Adam Lucas, Simon Naylor

**Affiliations:** School of Geographical and Earth Sciences, University of Glasgow, Glasgow G12 8QQ, UK; School of Geographical and Earth Sciences, University of Glasgow, Glasgow G12 8QQ, UK; Department of History, Heritage and Global Cultures, Nottingham Trent University, Nottingham NG1 4FQ, UK; School of Humanities and Social Inquiry, University of Wollongong, Wollongong, NSW 2522, Australia; School of Geographical and Earth Sciences, University of Glasgow, Glasgow G12 8QQ, UK

**Keywords:** Industrial Revolution, geomorphology, waterpower, mills, steam engines

## Abstract

The Industrial Revolution precipitated a pivotal shift from waterpower to coal-fueled steam power in British textile mills. Although it is now widely accepted that steam was chosen to power factories despite the availability of sufficient waterpower resources across most of Britain, the location and suitability of that waterpower during the early 19th century remain underexplored. Here, we employ quantitative fluvial geomorphology alongside historical climate data, factory records, and a catalog of over 26,000 mill sites to reveal that waterpower was abundant for most of early 19th century Britain, except in the central hub of British cotton production: Greater Manchester in the Mersey Basin. Our findings show that surging factory mechanization and overcrowding on key waterways in the Mersey Basin compounded waterpower scarcity arising from a drier 19th century climate. Widespread adoption of coal-fueled steam engines in certain key industrial centers of Britain was a strategy aimed at ameliorating some of the reduced reliability of waterpower. The fact that steam engines were frequently used in water-powered factories in many industrial regions until the third quarter of the 19th century to recirculate water to provide that power, or as a power supplement when waterpower availability was restricted, adds further weight to our argument. Rapid adoption of coal-powered steam engines reshaped the social and structural landscape of industrial work, firmly established Britain's prominence as an industrial powerhouse, and had lasting global industrial and environmental impacts.

**Significance Statement**

The First Industrial Revolution took place in Britain and was marked by profound socioeconomic and technological changes, including widespread adoption of coal-powered steam engines. Prior to this, waterpower was used extensively for nearly 2,000 years, primarily for grinding grain. Historians disagree about how much waterpower was available to manufacturers, but we show that in the heartland of British “Cottonopolis” around Greater Manchester, it was not as abundant as previously assumed. Limited waterpower drove factories to adopt steam engines to first assist, and then replace, waterpower as power needs escalated in the early 19th century. The transition to steam-powered cotton factories reshaped British industry and left a lasting global impact through the widespread use of fossil fuels for mass production.

## Introduction

Rapid industrialization had its origins in mid- to late-18th century Britain, particularly following the widespread mechanization of water-powered textile factories between ∼1770 and 1840 CE (1, 2). By the mid-19th century, Britain housed over 69% of the world's spindles and was the dominant global producer of finished cotton textiles (3, 4). Despite nearly two millennia of application in Britain (5–8), waterpower became increasingly supplanted in the textile industries by coal-powered steam engines over the same interval (9–11). This widespread shift from water to steam power in early factories, which we refer to as the “water-to-steam transition”, had dramatic long-term socioeconomic and environmental impacts (12–15), including irrevocable alterations to the Earth's climate and biosphere (16, 17).

The causes of the water-to-steam transition remain highly contested. Although it is likely that a combination of factors led to the relative demise of traditional water-powered industry to that powered by steam in Britain (2, 4, 13, 18), the role of the physical environment, and specifically the continuing availability of waterpower in this transition, has not been fully explored (19–21). In this article, we investigate the hypothesis that limited waterpower contributed to a rise in the use of coal-powered steam engines in early textile factories in Britain. This view was first articulated in a series of late 18th and early 19th century commentaries (22) (Supplementary Appendix) and has continued to influence debates since.

Using the first historical waterpower potential datasets for the entirety of Britain at 50-m resolution, we demonstrate that in the geographic heart of the British Industrial Revolution and first global center of mass production—the “Cottonopolis” of Greater Manchester (23)—historical waterpower was neither as ubiquitous nor as abundant as previously suggested. By integrating waterpower data with archival factory reports, we argue that increasingly scarce waterpower resources in the industrial heartland of NW England during the early 19th century may have compelled manufacturers to adopt steam engines, not only to guarantee necessary power requirements for production, but to prevent any limit to future growth.

**The water-to-steam power transition.** Britain's textile industry has often been used to exemplify the progress of 18–19th century industrialization. Textile mills experienced rapid growth during this period, becoming the first widespread factories, and were the primary users of industrial waterpower before becoming some of the earliest adopters of steam power (1). Arguments for when and why this transition occurred can be organized into four schools of thought, each of which place different emphases on the relevant evidence as to whether it was (i) an energy bottleneck or “crisis” (13, 24–28), (ii) the diffusion of steam engine and textile technology (28–31), and/or (iii) control over labor, production costs, and profitability (14, 18, 30) that primarily stimulated the transition.^a^

As early as 1835 CE, cotton historian and steam enthusiast Sir Edward Baines (22) argued that a scarcity of waterpower would have hindered British industrial growth after 1790 CE if not for the increasing adoption of steam power. His argument found wide support among his contemporaries (32) (Supplementary Appendix). Some 20th century historians supported Baines’ argument, suggesting that limited waterpower would have triggered a British “energy crisis” by the 19th century if not for the transformative implementation and “perfection” of the steam engine (13, 24–28). Exploiting British coalfields to power machinery unlocked an apparently limitless and arguably cheaper source of energy for industrial growth. This view has since lost explanatory traction among those who favor a slower model for the diffusion of steam power technology (2, 10, 33–35). This slower model accords with earlier arguments that waterpower remained more important than steam power until the second quarter of the 19th century (36–38). Informed by factory, insurance and patent records, these more recent scholars favoring delayed diffusion of steam technology instead suggest industrial waterpower remained significant until at least the mid-19th century, relying on evidence that waterpower potential remained underutilized even in the most industrialized regions of Britain (39, 40). Renewed consideration of costs associated with water-powered and steam-powered mills through the 19th century indicate that in the long-term, water power was as cheap, if not cheaper than, steam power in many cases (c.f., Refs (2, 4, 10, 41, 42)).

This argument concerning a much slower rate of diffusion with respect to steam power technology raises significant (and as-yet unresolved) questions about why and where industrialists adopted steam engines, if waterpower was not only available but no more expensive than steam power. On the one hand, it has been suggested that steam engines provided a wealth of spatiotemporal and political advantages (4, 10, 18), once improvements to early engine models were implemented (29, 41, 43). Waterpower was site-dependent and thus comparatively immobile, finite, and, by its very nature, irregular (20, 44, 45). Steam engines, on the other hand, arguably allowed textile manufacturers to bring down labor costs (30), and to situate themselves more advantageously in relation to existing pools of labor and skilled artisans (4, 29, 31). Some argue that, by adopting steam, mill owners could continue to maximize their profits while better negotiating increasing restrictions on working hours imposed under the 19th century Factory Acts (14, 18, 46, 47). Despite these different arguments, the only consensus seems to be that no one socioeconomic variable, including comparative per unit energy costs, can fully explain the transition to steam. Nonetheless, nearly all explanatory models rely on biased observations of waterfalls and waterpower in their interpretations, often at an insufficient spatial or inappropriate temporal scale to conclusively address the role of the physical environment during this energy transition.

**Fig. 1.**
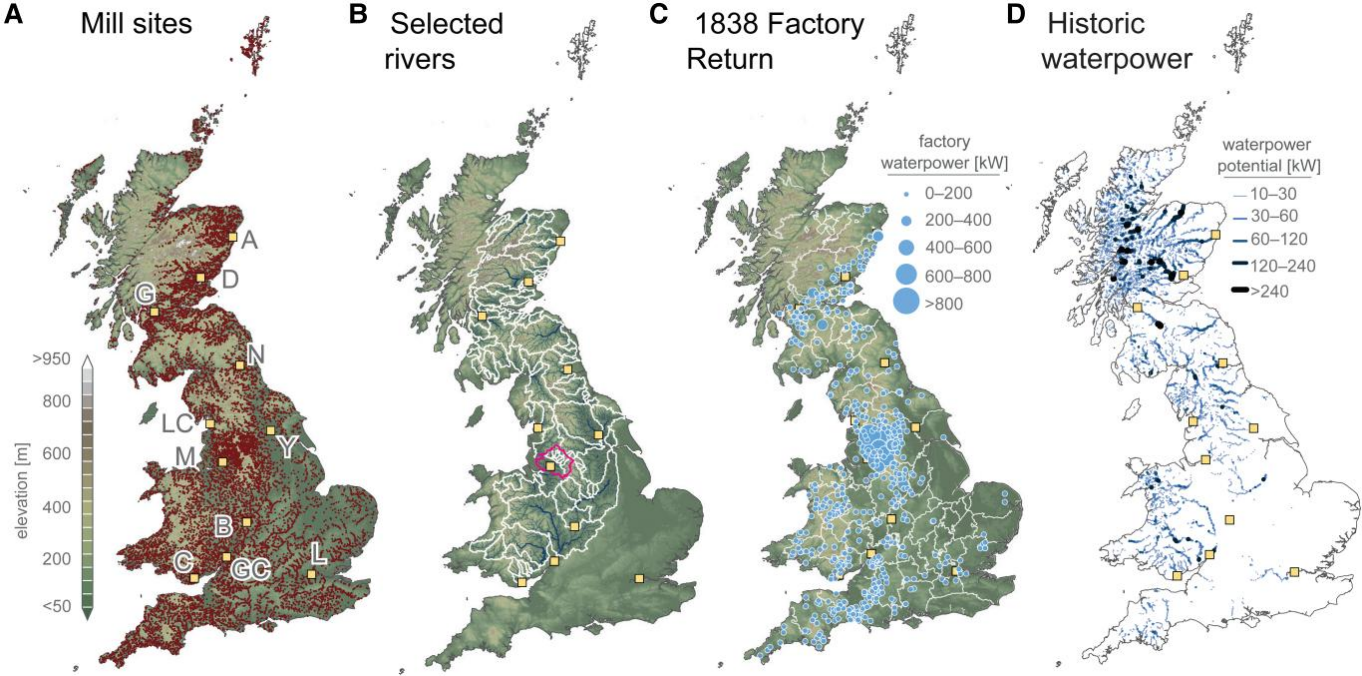
Historic mill sites, rivers, and waterpower. A) Mapped mill records (*n* = 22,621) from 1729‒1836 CE for all British historical counties (Materials and methods), compared to B, rivers (graduated lines) and river basins (outlines), with the “Cottonopolis” of the Upper Mersey Basin shown with darker magenta outline; C) reported waterpower from the complete 1838 Factory Return (48) for major textiles of cotton, wool, silk, and worsted with historic county boundaries (outlines); and D) calculated raw theoretical historical waterpower potential. Major city centers as squares. A, Aberdeen; B, Birmingham; C, Cardiff; D, Dundee; G, Glasgow; GC, Gloucester; L, London; LC, Lancaster; M, Manchester; N, Newcastle; Y, York.

**Waterpower and a British energy crisis.** The evidence for limited waterpower driving a potential British energy crisis in the early decades of the Industrial Revolution originates mainly from enthusiasts for steam power such as Baines (22) (Supplementary Appendix), and is based almost exclusively on secondary evidence about textile manufacture on the Pennine rivers of England (Fig. 1A). Historians often suggest that the scarcity of waterfalls hampered the expansion of historical waterpower generation in the British textile industries (2, 25, 26), often unfairly compared to the East Coast American textile industries and the 1400-km long “Fall Line” escarpment (49). Such a view overlooks the fact that large waterfalls were not always required to generate sufficient power for textile factories (50, 51), and that weirs functioned as effective artificial waterfalls for centuries in Britain (6, 7, 52). Geomorphological evidence further indicates that many British mills utilized a broader range of river knickpoints than previously recognized in the economic history literature; individual step-wise knickpoints (i.e. waterfalls) were not the only geographic features exploited to maximize waterpower generation from steep reaches of river (50, 53–56). Furthermore, the co-location of mills across even broad knickpoints questions arguments that correlate mill crowding with limited waterpower (c.f., Refs (1, 2, 10, 22, 57). Such a correlation relies on several circular assumptions concerning mill competition and local waterpower availability; often concluding that dense congregations of watermills must have competed over river water, which in turn must have been limited in terms of waterpower due to the aforementioned overcrowding. These correlations and assumptions frequently underpin much of the observational evidence presented by 19th century contemporaries concerning mill crowding, and subsequently served to inform the energy crisis hypothesis developed by some 20th century historians (58) (Supplementary Appendix). However, in the occurrence of broad, km-scale river knickpoints, historic crowding of watermills may just as well indicate a strategic choice by mill owners to occupy a river reach with exceptional (rather than limited) waterpower potential. Consequently, mill numbers along lengths of river should not be directly correlated to waterpower potential without geomorphological context, or unless direct evidence from millers on the river in question is available (44, 45).

**Previous assessments of waterpower potential.** Only two prior studies (39, 40) attempted to test whether waterpower rather than steam power could have fulfilled the needs of the early textile industries. By comparing estimates of natural waterpower availability to the amount of waterpower required for manufacturing reported by textile mills in 1838 CE (48), these studies argued that only as much as 7% of waterpower resources were ever fully utilized in Britain, even in the most heavily industrialized regions of NW England. It was suggested that socioeconomic and technological factors alone, not limited waterpower, incentivized the shift to steam power (40). Whether such results and conclusions are an accurate representation of the British water-to-steam transition or effectively debunk the energy crisis hypothesis remain debatable. These studies (39, 40) used limited topographic and meteorological data at coarse resolution from NW England alone to estimate historical waterpower, and their conclusions relied on incomplete factory reports from only some textile industries. Other studies have since used modern flow data aggregated to the county level in attempts to parameterize water availability (4, 31), but many modern British rivers have unnatural flows subject to urban abstraction and base flow management that bias direct comparisons with the historic period (59).

Below we evaluate the availability of waterpower resources across mainland Britain during the water-to-steam transition (∼1770‒1840 CE). To assess if and where limited waterpower may have driven textile manufacturers to seek alternative sources of power, we generate high-resolution waterpower potential datasets representative of early 19th century Britain and compare our results against complete factory reports for steam- and waterpower usage across all major English, Scottish and Welsh textile industries (48). Our waterpower potential estimates draw on quantitative geomorphological flow-routing techniques informed by 19th century climate and meteorological observations (Materials and methods) to generate river discharge and waterpower potential datasets. We further correct our waterpower potential dataset for historic land-use following point pattern analysis of mill sites compiled from over 40,000 records from early county maps (Fig. 1) and for the artifacts imposed by over 47,000 post-industrial waterway barriers built during the intervening period.

## Results

### Geomorphic controls on waterpower potential

We first assess where historic mills were most often cited by conducting point pattern analysis on all 1729‒1836 CE sites recorded in our cartographic mill census (*n* = 15,570; Materials and methods). Results show that nearly one-third of the British landmass was rarely used for historical waterpower generation, with ∼90% of mills sited below 200 m asl. (Figs. 1C and S1). Less than 1% of mills were cited above the 300 m asl contour, broadly coinciding with the transition from more agriculturally productive land to upland peat- and moorland (Supplementary Appendix). We term this stark elevational demarcation and the absence of mills above ∼300 m asl the “Mill Line”, above and within which exceedingly few mills of any type were historically sited in Britain. Further analysis of watermill sites surveyed before 1838 CE (*n* = 11,849) suggests that the combined increase in hillslope gradient adjacent to river channels and decrease in contributing river drainage area with elevation made siting above 200 m asl unfavorable and severely disadvantageous above 300 m asl (Materials and methods; Fig. S5). Given the long history of mills in Britain and their importance to local communities (5–8), we propose that locations of settlements, transport networks, and mill sites on river terraces above moderately powerful river reaches and within more agriculturally productive land (60) reinforced this spatial co-dependence over time. Therefore, we adjust our waterpower potential calculations to reflect only the regions most likely to have been used for historical waterpower generation by mills (Materials and methods; Data S1).

### Waterpower availability

Early 19th century waterpower potential estimates are much less than predicted by earlier studies. Even when our theoretical waterpower potential estimates are left uncorrected for historic land-use and for apparent falls induced by modern dams and spillways (Figs. S2 and S3; Tables S1–S4), our estimates are, on average, only 29‒44% of earlier estimates (Table S1) (40). We attribute this considerable difference to recent technological advances in the fields of geomorphology and meteorology, namely through the availability of high-resolution gridded topographic and moisture data built from satellite observations and calibrated by meteorological gauging (Materials and methods). Prior studies (39, 40) relied on more limited topographic and meteorological data available at a much lower resolution than the 50-m/pixel resolution utilized here. When we further correct theoretical waterpower potential datasets for land-use and waterway obstructions, the greatest reduction in waterpower potential is due to historic land-use. If upland areas atypical for historic mill sites (>300 m asl, peatland) are not excluded from analyses, waterpower potential can be overpredicted by 23‒69% (average 41%; Fig. S2; Tables S3 and S4).

**Fig. 2.**
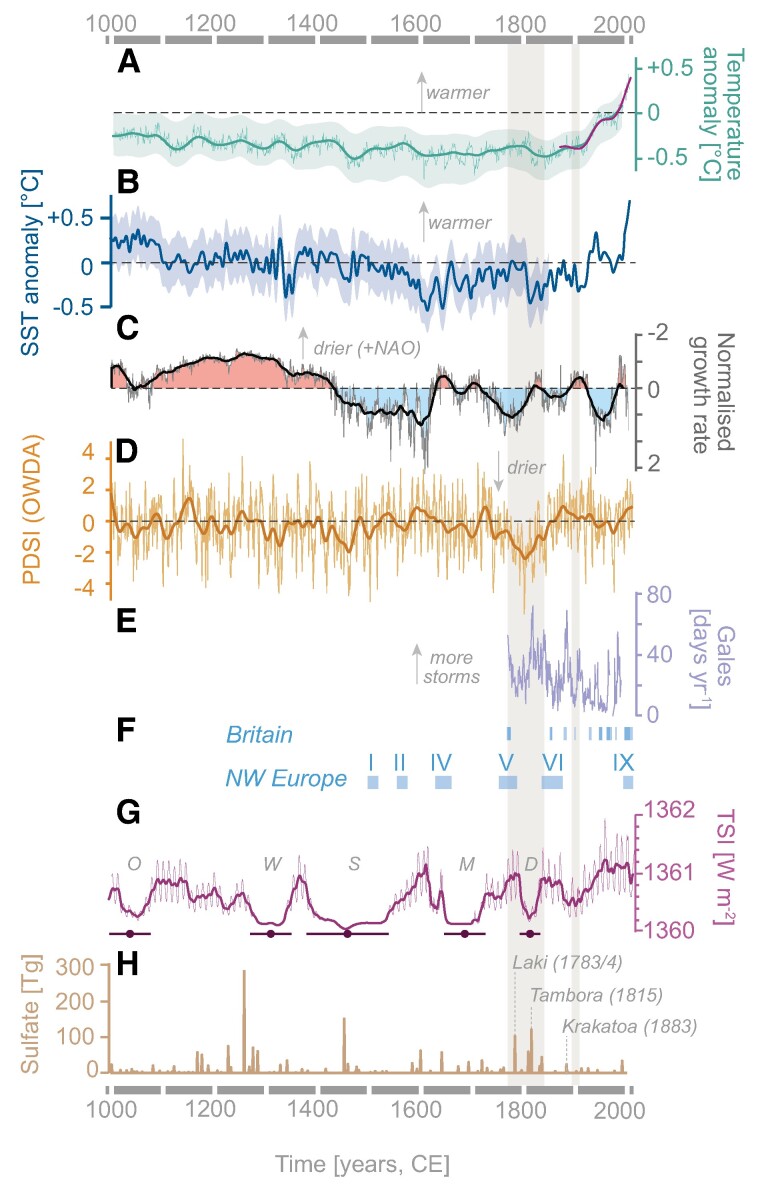
Climate variability over the last millennium. A) Global mean surface temperature anomalies and ensemble median subjected to 31-year bandpass filter from the Coupled Model Intercomparison Project (61), with respect to 1961‒1990 CE. Target HadCRUT4 (62) instrumental data from 1850‒2017 CE subjected to 31-year bandpass filter (purple line). Envelope indicates 31-year filtered 2.5th and 97.5th percentiles of full ensemble. B) Decadal sea surface temperature (SST) anomalies reconstructed over the North Atlantic (63) as a proxy for Atlantic Multidecadal Oscillation (AMO) variability. Envelope indicates 95% confidence intervals. C) Proxy record of winter January–March precipitation tightly correlated to North Atlantic Oscillation (NAO) variability shown by the inverted composite stalagmite record from NW Scotland (Uamh an Tartair) (64) with drier (positive NAO, red) and wetter (negative NAO, blue) phases indicated on the 20-year average. D) Regional Old World Drought Atlas (OWDA) Palmer Drought Severity Index (PDSI) for June–August (65) across the British Isles (−11°E‒−2.25°E, 50°N‒61°N) for 1000‒2012 CE, with 31-year mean spline. June–August PDSI reflects spring–summer soil moisture conditions. E) Winter storminess from 1780‒1988 CE as indicated by annual gale-day frequency in January–December at Edinburgh, Scotland (66). F) Flood-rich periods in Britain (67, 68) and NW Europe (69). G) Annual total solar irradiance (TSI) (70) with grand sunspot minima (71). Grand minima: D, Dalton; M, Maunder; O, Oort; S, Spörer; W, Wulf. H) Annual global stratospheric volcanic sulfate aerosol injections from 1000‒2000 CE, with major 18‒19th century eruptions (72, 73). Vertical beige bars are 1770‒1840 CE and 1891‒1910 CE intervals.

We further consider the role of historic moisture on waterpower potential estimates (Fig. 2). Paleoclimatic data (62–65, 74, 75), climate ensemble models (61) and early hydro-meteorological evidence from rivers and canals (67–69, 76, 77) suggest the early 19th century was one of the coldest and driest intervals over the last millennium across the British Isles and Europe (Supplemental Text). Yet limited direct measurements of precipitation and temperature exist in Britain before systematic observations began in 1862 and 1891 CE (78), respectively. Therefore, while we consider several moisture scenarios from the 1891‒2015 CE gridded meteorological time series for temperature and precipitation in our analyses (Materials and methods), we argue that the first decade of observations (1891‒1901 CE) offers the most reasonable proxy for the early industrial era (1770‒1840 CE) due to similar solar (70, 71), oceanic (64, 74), and regional volcanic forcing (72, 73) conditions occurring before significant industrial warming (>0.5°C) (79–82). When broadly wetter modern (1961‒2015 CE) moisture conditions are applied rather than the comparatively dry early industrial conditions, waterpower potential is overpredicted on average by ∼12‒13% (Fig. S2).

**Fig. 3.**
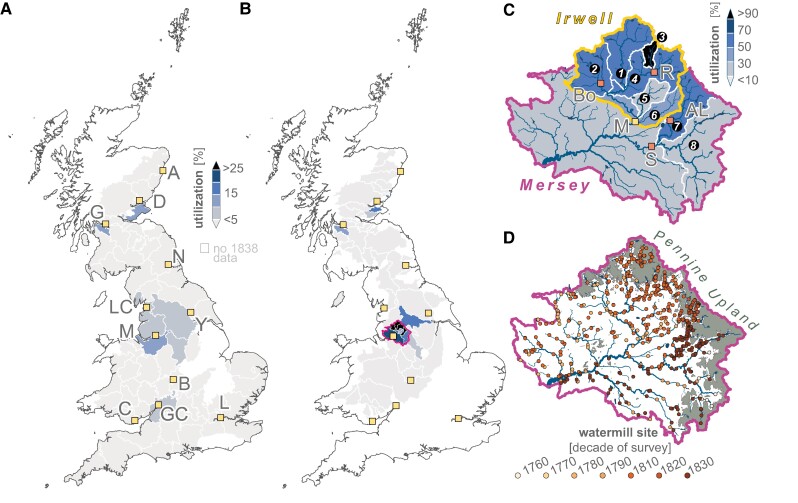
High utilization of early 19th century waterpower in British “Cottonopolis”. A) Waterpower utilization by historical county (Table S3). B) Waterpower utilization by river basin (Table S4). Upper Mersey Basin outlined in magenta. C) Waterpower utilization of the Upper Mersey Basin and its tributary catchments, including the River Irwell (inner yellow outline): 1—Upper Irwell, 2—Croal, 3—Roch, 5—Irk, 6—Medlock, 7—Tame, 8—Etherow-Goyt. D) Upper Mersey Basin mills (*n* = 543 watermill sites; 1729‒1836 CE) colored by decade of map survey, with land above “Mill Line” highlighted in dark gray. Efficiency of 40% assumed for waterpower generation (Materials and methods). Major city centers as yellow squares in A and B after Fig. 1, with textiles centers of Greater Manchester mentioned in text as orange squares in C. AL, Ashton-under-Lyne; Bo, Bolton; R, Rochdale; S, Stockport.

**Waterpower utilization.** Due to growing concerns about the working conditions for women and children in textile factories in the early 19th century, a series of Factory Acts initiated mass inspection of textile mills across Britain (46, 47), including systematic collection of statistical data on the types and magnitudes of motive power used in each factory (83). We compiled the earliest comprehensive report publishing this data, the 1838 Factory Return (48), to indicate waterpower demand required by the textile industries (Fig. 1C and Table S5). When natural waterpower availability is directly compared to textile factory waterpower demand, the proportion of waterpower utilized indicates that no historical county was using more than 14% of available waterpower (Fig. 3 and Table S3). Most counties utilized less than 2% of their waterpower resources. Results would suggest that neither early industrial England, Scotland nor Wales fully utilized their waterpower potential. Findings at the county-scale broadly corroborate earlier arguments that no industrialized textile area fully utilized its potential waterpower (39, 40). Yet when waterpower availability is reviewed against factory waterpower use at the basin-scale, high levels of utilization are clearly identifiable (Fig. 3B and Table S4). If watermills are assumed to generate power by abstracting water “run-of-the-river” with breast- or overshot waterwheels operating in total at 40% efficiency (40), up to 26% utilization of waterpower is observed, on average, by the Greater Manchester textile industry that was spread across the Mersey Basin (4,455 km^2^).

The majority of waterpower use in the Mersey Basin occurred on the River Irwell (707 km^2^; Fig. 3C, D). The Irwell reached 40% utilization of its waterpower potential on average, with the Upper Irwell, Croal, and Spodden tributaries (26‒167 km^2^) reaching upwards of 41‒153% utilization if all mills are assumed to be in operation at any one time (Table S4). Up to half of each basin lies above the “Mill Line”, with no recorded mills above 300 m asl in any of these basins according to early county maps. This stark geomorphic threshold, if used to correct raw theoretical waterpower estimates, reduces the historically exploitable waterpower potential on average by ∼55%. Furthermore, even if all river discharge is assumed to be abstracted to mills for power generation and converted with 100% efficiency, the entire River Irwell remains 15% utilized in terms of textile factory waterpower usage alone in 1838 CE. Even in such an unlikely scenario, the Spodden tributary outside Rochdale reaches nearly 57% waterpower utilization. Similarly, other tributaries, including the Tame, Etherow, and Medlock feeding the satellite industrial centers of Stockport and Ashton-under-Lyne all reach 16‒38% average utilization. In Scotland, utilization west of Glasgow reached 3‒11% in the cotton-spinning nexus of the River Cart (567 km^2^) and 2‒17% in the eastern flax-spinning centers at Dundee and in Fifeshire.

### Impact on the British textile industry

Results here highlight that while waterpower remained available across most British counties and river basins in 1838 CE, scarcity emerged where localized, growing industrial demand outstripped the natural waterpower availability. Claims of a nationwide energy crisis triggered by waterpower scarcity (13, 24–28) are largely unfounded. On the contrary, at the *national* level in 1838 CE, waterpower resources remained significantly underutilized even 60 years into the classical period of the Industrial Revolution (∼1770‒1890 CE). Run-of-the-river waterpower was amply sufficient to fulfil most power demands reported by the 1838 Factory Return, even if waterpower were to substitute for power generated by steam engines (Fig. S4).

However, in the major cotton textile production region north and east of Manchester, increasingly limited run-of-the-river waterpower is clear by 1838 CE (Fig. 3b; and Table S4). First dubbed “Cottonopolis” in 1854 CE (23), Greater Manchester occupied the same highly industrialized but underpowered tributaries (Upper Irwell, Tame, Spodden) discussed in the early narratives that informed more recent claims of a British energy crisis (Supplementary Appendix). Even with generous assumptions for waterpower availability and energy efficiency, most catchments powering the growing industrial Cottonopolis utilized more than 24% of naturally available waterpower. Use of above 24% theoretical waterpower potential capacity, especially above ∼40%, exponentially increases the relative marginal costs to further develop hydropower in a watershed, in part due to increasing water conflict (40, 84, 85). Although exact thresholds are difficult to constrain for each historic basin (Supplementary Appendix), the fact that almost every Greater Manchester basin exceeded modern cost thresholds illustrates the intensity of energy utilization over the time period in question. The post-glacial topography and extensive moorland of Pennine rivers like the Irwell first encouraged concentration of water-powered mills along steep river corridors. This morphology also restricted the later expansion of watermills farther into the available catchment far above 200 m asl. Our findings lend credence to historical accounts regarding waterpower scarcity and the necessity for additional motive power to fuel Cottonopolis (22, 32). The modest physical advantages that first allowed water-powered industry to emerge on these rivers were swiftly surpassed in the early 19th century. Increasing mechanization and textile mill size (48, 86, 87) pushed total power demand beyond the natural capacity of waterpower resources in and around Greater Manchester. This was not the case, however, in most of the other industrial centers throughout Britain.

While our work primarily assesses waterpower availability, it also highlights the rising importance of the provision of sustained, regular power to early factories. As the size and the number of machines working simultaneously for spinning and weaving increased (1, 22, 87, 88), the demand for a consistent and continuous power supply also grew more pronounced (89). Waterpower is inherently variable and a function of river discharge; the greater the discharge required, the more frequent the interruptions to the flow will be. In all but the most exceptional locations, sustaining high levels of waterpower requires regulating infrastructure. Historically, the most common and affordable way to regulate waterpower was through construction of mill ponds, such as are regularly depicted by late 18th century cartographers (Fig. S5). By the 1790s, the majority of Scottish basins with over 5% waterpower utilization used artificial lochs and reservoirs to support mills, with several complex waterwork schemes engineered specifically for water-powered milling by the 1820s (35, 90). Overall, however, few large reservoirs or water engineering schemes were constructed in the first quarter of the 19th century for water-powered milling in Britain (10, 91). Instead, many of the first reservoirs were purpose-built to supply water for the expanding arterial canal network between 1790 and 1820 CE (52, 92, 93), not to power watermills nor to provide clean water for rapidly urbanizing populations. Yet, while it is plausible that canals effectively curtailed construction of similarly sized reservoirs for watermills, textile manufacturers relying on waterpower were often instrumental in passing the legislation necessary to build canal infrastructure (94). The benefit to mills could be manifold. Canals often provided power for watermills but, as navigable waterways, they primarily expanded the domestic market and expedited the transport of coal at a greater volume across the country (95).

As early as 1765 in England and 1791 CE in Scotland, an alternative solution was employed to regulate waterpower: the recirculating steam engine (34, 90, 96). Contrary to those views that assume that steam power simply replaced waterpower throughout industry, recent examination of evidence contained in steam engine sale records, the Factory Acts and factory inquiries reveals that steam engines were widely used to recirculate water for some factories and other industries reliant on hydropower well into the latter part of the 19th century (34, 45, 96). Almost as early as mechanized textile manufacture began in Britain, coal-fired steam engines were first employed as assistants to waterwheels. At least 213 recirculating engines in various industries are known to have first returned water directly to a waterwheel or to a mill pond by the start of the 19th century in Britain (96). Of all the known water returning engines associated with cotton mills, 24 of 64 (∼37%) were adopted at sites in the Upper Mersey Basin: within lowland central Cottonopolis and small upland tributaries of the Upper Irwell, Roch, Croal, and Tame (Table S6). By installing hybrid motive power systems, mill owners could better regulate available waterpower at all times and seasons. Increasing restrictions on working periods for children and women after 1819 CE for cotton mills and especially after 1833 CE for all textile mills (45–47) made operation during legislated hours of tantamount importance. Increasingly deprived of the ability to make up hours, it is not difficult to surmise why textile manufacturers took advantage of increasingly available technologies and resources at hand. No relocation was required and time efficiency could thus be maximized at the same site (14, 18). Moving elsewhere to more abundant waterpower resources would have been commercially and industrially impractical; connections to established factory labor pools, skilled artisans, and markets were also vital considerations for business (4). The adoption of an additional motive power source could meet local industrial demands, mitigate the effects of increasingly restrictive factory legislation, and remove limits to future business growth.

In summary, our findings reconcile certain aspects of leading explanatory models for evolution of the British textile industry: the British “energy crisis”, the diffusion of steam engine and textile technology, and a growing shift towards capitalistic control over time and productivity. While much of Britain's waterpower resources remained underutilized, Greater Manchester's Cottonopolis experienced waterpower scarcity. While it is possible that drier 19th century climate conditions across the British Isles limited waterpower availability (11‒23% regional reduction in waterpower potential), even if all available discharge was used for hydropower generation at 100% efficiency under modern-day moisture conditions, a remarkably similar conclusion would be obtained (Table S4). We therefore argue that limited waterpower likely acted as a direct, physical catalyst incentivizing the adoption of steam power by manufacturers in Britain's Cottonopolis. They were unwilling to relocate, unable to improve regulatory water infrastructure, and looking to maximize production during available working hours under increasingly restrictive factory legislation. The locations at which the earliest engines were deployed in the textile industry highlights that there was already appreciation by factory owners of the limitations that waterpower presented in the region around Greater Manchester. This prompted the widespread adoption of recirculating engines to first assist what limited waterpower that was available from as early as 1783 CE in the Mersey Basin.

Continued growth into the mid-19th century and further concentration of cotton textile production into the region around Greater Manchester (4, 97) stimulated rapid adoption of coal-powered rotary steam engines for cotton spinning and driving other textile machinery. This entrenched social and structural transformations in work patterns and the division of labor now considered emblematic of Britain's Cottonopolis and the first Industrial Revolution. Outside of and in contrast to the Mersey Basin, waterpower tipping points were not reached until later in the century, if at all (34, 35). Our integration of geomorphological and climatic datasets with compiled cartographic and archival evidence invites careful reconsideration of resource allocation relative to the rapid development of new socioeconomic interests and technologies during the industrial era. This is especially important with regard to the displacement of pre-existing renewable water- and wind power technologies in Britain and beyond; technologies that continued to be developed throughout the 19th century (4, 9, 49, 89, 98, 99). The fact that water continued to be harnessed as the predominant source of motive power in hybrid power systems of varying sizes and applications elsewhere in British industry until at least the 1870s (96) further undermines arguments for a wider British energy crisis. Our research demonstrates that while limited waterpower existed in and around Cottonopolis, other causal factors are required to explain widespread adoption of steam power in other industrial centers during the British Industrial Revolution.

## Materials and methods

### Theoretical waterpower potential

The moisture balance in any natural watershed is controlled by the delivery of water as precipitation (*P*) and the loss of water through evapotranspiration (ET) and seepage as long-term groundwater infiltration (*G*) and variable short-term water storage (*S_w_*) over an entire watershed area (*A_w_*). The net water remaining, called runoff, flows down gradient to physically manifest as river discharge (*Q*, m^3^ s^−1^):


(1) $$Q=A_{w}(P-\mathrm{ET}\pm G\pm S_{w}).$$

Although the entirety of the gross river discharge (*Q*) has the capacity to do work, the actual discharge diverted for hydropower generation will never equal the total river discharge. The potential power (*P*, Watts or J s^−1^) generated by a hydropower structure, such as a waterwheel, depends on (1) the portion of the discharge utilized for power generation (*Q_d_*), (2) the net fall, or head, of water (*Δh*) assessed for the specific site and (3) the net efficiency of the entire system (*η*, %). This forms the basis of the generic hydropower equation (100) that describes how power can be generated by the energy of flowing water falling from height:


(2) $$P=\eta \cdot \rho \cdot g\cdot Q_{d}\cdot \Delta h$$

where *ρ* is the density of water (1,000 kg m^−3^) and g is the acceleration due to gravity (9.81 m s^−2^). The power potential can thus be considered the total power hypothetically possible at any site, with assumptions for net efficiency and design discharge (*Q_d_*). The net head (*Δh*) is a site-specific factor, often calculated as the total height difference between the constructed mill lade or dam and the base of the hydropower structure (i.e. waterwheel). Historically this is why many watermills tend to be located immediately downstream of bedrock knickpoints, such as waterfalls or over-steep reaches of river, because fall can be maximized over a minimal along-stream distance and at reduced cost (50, 54, 55). Here we assume a run-of-the-river outlay common for mills across Britain (19, 50, 101), where river water is diverted to a vertical-wheeled watermill from the river by sluice to a mill lade (or leat) and water is returned from the wheel back to the river by a tail race. We follow prior studies applying a conservative 40% efficiency for the combined portion of the discharge abstracted to a vertical-wheeled watermill equipped with an overshot or breastshot waterwheel (40, 51, 102).

Historical waterpower potential is derived across Britain (Tables S3 and S4; Data S1) using standard flow routing techniques over a hydrologically consistent digital terrain model (DTM) weighted by gridded historical runoff. We utilize the updated 2016 UK Centre for Ecology and Hydrology (CEH) 50-m Integrated Hydrological DTM (IHDTM)(103) consistent with the CEH 1:50 K digital river network (104). Prior to network delineation by flow routing techniques, the IHDTM was “burned-in” by 1 m with the CEH 1:50K digital river network (>0.5 km^2^ threshold drainage area) and iteratively preprocessed (105) to remove any remaining elevation artifacts using MATLAB-based TopoToolbox 2 (106) and Topographic Analysis Kit (107). Constrained regularized smoothing of the longitudinal profile applied a stiffness constant of *K* = 10 and tolerance of *τ* = 0.2 (105). Fall, or head, of the river was calculated as the elevation difference between each river channel cell node and the subsequent downstream node (∼50‒70 m horizontal distance). The resolution and cell-to-cell flow routing methods employed here for assessing hydropower potential are consistent with methods employed by government agencies (84, 108) and recent global studies (109, 110).

### Exploitable waterpower potential

Gross theoretical power potential, while informative, is of limited practical value since local physical factors (landcover, hillslope, contributing drainage area), existing infrastructure, and the cost and capacity of hydropower technology (i.e. waterwheel type) all affect how much waterpower can realistically be generated at any one site and time (110, 111). Raw theoretical waterpower potential estimates (Data S1) were corrected for post-industrial waterway obstructions and regions not used for power generation by textile mills, such as lake, canal, and tidal waterways (Supplementary Appendix). We further adjusted estimates by excluding land rarely used by historical mills in Britain: regions above 300 m asl and land broadly classified as moor- and peatland (112), following observations in this study and from the literature (35). Theoretical (Fig. 1D) and exploitable power potential are presented for Britain, summarized by historical county as well as for 112 industrial river basins (Fig. 3A and B; Tables S3 and S4; Data S1).

### Historical climate and moisture

Limited precipitation and few direct observations of temperature exist before systematic observations initiated in 1862 and 1891 CE, respectively. We therefore utilize gridded systematic observations from the 1891‒2015 CE timeseries (113–116) to assess waterpower in Britain from four intervals: (i) early historical (1891‒1901 CE), (ii) long-term historical (1891‒1940 CE), (iii) series average (1891‒2015 CE), and (iv) modern (1961‒2015 CE). Respectively, these intervals broadly represent: a drier historical interval before significant modern warming (+0.5 °C (81)); a long-term interval before significant modern warming; the series average; and a modern interval subject to significant modern warming. While all moisture scenarios are considered in our analyses (Tables S3 and S4), we present the first moisture interval (1891‒1901 CE) as the most representative proxy for early industrial-era (1770‒1840 CE) moisture conditions. This follows historical archival evidence, paleoclimatic data and ensemble models for the United Kingdom, North Atlantic, and Europe over the last millennium (Fig. 2; Supplementary Text).

We use 1-km gridded HadUK monthly precipitation (115) and the Historic Droughts 5-km monthly potential evapotranspiration (PET) (113, 116) datasets. Monthly meteorological data were annually aggregated in QGIS 3.16 and used to derive downscaled (50-m) runoff following a simple moisture balance (precipitation minus potential evapotranspiration; P-PET) relationship for weighting in waterpower potential calculations.

### Historical waterpower use

Due to growing concerns over working conditions and hours for women and children in textile mills, implementation of the Factory Acts after 1833 produced a series of statistical reports on employment, working hours and power usage. The 1838 Factory Return, which recorded data at the national, county and sub-regional level (48), is widely considered to be the earliest comprehensive report of steam- and waterpower in textile mills for Britain (2, 10, 34, 83). From this Factory Return, we use installed waterpower capacity as a generous proxy for waterpower in-use, or waterpower demand. Waterpower demand was compiled for the major cotton, worsted, wool, flax, and silk textile sectors, converted to SI units (1 hp = 745.69987 Watts), and georeferenced to the nearest reported civil administrative center (Fig. 1C; Table S5). Waterpower utilization is calculated as the ratio between calculated natural “run-of-the-river” waterpower potential and reported waterpower use by early textile factories aggregated at the county or basin level.

### Cartographic mill census

Although it has been claimed that 20,000 mills existed in Great Britain by the early industrial period (19), no central or public register of industrial-era mills exists. Here we present the first georeferenced 1729‒1914 CE cartographic mill database for Britain. Map coverage exists for almost all historical Scottish, English and Welsh counties, including Cheshire, Lancashire, and Yorkshire, which are widely considered to be the most industrialized areas of Britain for the interval studied here.

We record 22,621 water- and steam mill records from British county, estate and military survey maps (Figs. 1, 3, and S5; Data S1). Locations, mill types, and power type, if indicated, were manually recorded from pre-Ordnance Survey map sheets held by the National Library of Scotland, the British Library, the National Library of Wales, the Royal Geographical Society (with the Institute of British Geographers), the National Library of France, the Digital Archive at McMaster University Library, the Yale University Library, and the David Rumsey Map Collection at the University of Stanford Library (Supplementary Appendix). Our cartographic work is further informed by other partial, nondigital mill censuses (34, 35) and the National Library of Scotland's “Scottish Water Mills project” developed from 1843‒1914 CE Ordnance Survey maps.

From maps, watermills were identified through a combination of graphical depiction, text, and locations relative to rivers: (i) waterwheel symbology; (ii) co-location with a depicted lade and/or tailrace; (iii) text indicating use as a watermill; (iv) geomorphic position typical to watermills (see Main text and Point pattern analysis); or (v) if within a 275-m buffer lateral to the delineated river network after Ref. (55). (Please refer to Supplementary Appendix for details concerning recording, correction, map scales, and accuracy.)

### Point pattern analysis of historic mill locations

Watermills by their very nature are innately dependent on the river networks that flow across landscapes. Objects associated with or constrained by a linear network, such as mill sites on rivers, can therefore be spatially analyzed through point pattern analysis, where site dependence on physical variables can be statistically evaluated relative to other observations and the network itself (117). While it is well-recognized that historical mill siting was dependent on numerous physical, technical, and social factors (4, 50, 55, 101, 118, 119), point pattern analysis here explicitly evaluates which topographic factors should be considered in context to historical county- and national-level hydropower generation. In particular, we primarily assess the role of elevation in limiting hydropower generation based on visual inspection of all mill sites (see Main text; Fig. 1) and earlier inference that watermills were rarely sited above 750 ft in Britain (∼228 m asl) (35).

From the cartographic mill census from 1729‒1914 CE (*n* = 40,893 records), we identify 26,458 unique sites after aggregating adjacent observations with a 70-m buffer (maximum downstream node length) in QGIS. Based on their proximity (<275 m orthogonal lateral distance) to the river network, we identify 19,725 unique sites that are either clearly defined as or are plausible watermills. From 1729‒1836 CE records (*n* = 23,621), we recognize 15,570 mill sites of all types, of which 14,321 records are clearly demarcated as watermills at 10,005 unique sites.

Mill sites from pre-1838 CE maps (*n* = 15,570) were determined to be inhomogeneously distributed (χ^2^-test score of *P* < 0.0001) when compared to a spatially random Poisson point distribution with homogeneous intensity simulated across the same landscape and stream network. This demonstrates that mill sites are not completely spatially random, and that spatial dependence on covariates could be further tested. Mill site dependence on elevation was then evaluated through comparison of mill empirical cumulative distribution functions (eCDFs) to eCDFs (*n* = 100,000 trials) of spatially random homogeneous Poisson point distributions simulated across the same landscape and river network. Greater than 90% of mills occur below 200 m asl, with ∼1% occurring above 300 m asl. Mills occur at slightly higher elevations than expected when below ∼50 m asl and at lower elevations than expected above ∼50 m asl, when compared to spatially random, homogeneously distributed points (Fig. S1a). This clustering of mills is further illustrated by the intensity $\left(\hat{\lambda }\right)$ of mills occurring at ∼50–200 m asl through nonparametric analysis of elevation (117, 120) (Fig. S4b).

To test how well elevation alone controls the spatial variation in pre-1838 CE watermill sites (*n* = 11,849 defined and likely sites), we fit a loglinear model to our dataset and used it to predict watermill intensities along the river network using an inhomogeneous Poisson point pattern (Fig. S1c). Although a binomial equation derived for elevation alone appears to simulate watermills reasonably well (*P*-value = 1.05 × 10^−255^), the area under the receiver-operating characteristic (ROC) curve indicates an AUC of 0.5922. Values close to one indicate perfect discrimination, where 0.6 > AUC > 0.5 indicate acceptable discrimination. This suggests that while elevation may partly explain the distribution of watermills, it cannot solely explain the distribution or clustering of all watermills. If mean upstream drainage area and mean hillslope gradient within 1,000 m of the river channel are further considered with elevation, the predicted intensity of watermills (Fig. S1c) and shape of the ROC curve with an AUC = 0.7422 (Fig. S1d) indicate good discrimination by our second model when compared to observed watermills.

Exploratory analysis here suggests that there exists shared physical characteristics among most watermill sites in Britain. Historic watermills are most often sited atop gently graded terraced floodplains and at lower elevations (50‒200 m asl) on moderately sized rivers. More explicitly, pre-1838 CE watermills are sited frequently on the downstream edge of abandoned river point bars, with mill lades stretching to the upstream edge of the point bar. Mills on small, low-order coastal streams are often collocated on knickpoints created from glacial isostatic adjustment and relative sea-level fall, as previously recorded in Scotland (55, 56). Small upland rivers with limited drainage areas and little to no terraced floodplain rarely host watermills. River size, floodplain extent and gradient, as well as drainage area, all covary to some extent with elevation, which together explain the first-order apparent control by elevation on mill sites during visual inspection.

Despite a reasonably good discrimination by our second exploratory model (0.8 > AUC > 0.7), we note that it still neglects to reproduce the full spatial distribution of watermills (e.g. AUC greater than 0.9) and a matching intensity curve. The empirical inhomogeneous K-function further underlines that watermill locations exhibit a stronger clustering than those simulated by our second model (Fig. S1e). We envisage this clustering is driven by other variables, such as earlier hand spinning and weaving centers, and early canal and road networks, that are not included in our landscape analysis. We recommend that analysis of such clustering requires dynamic evaluation of the distribution of mills by decade, function, and relative to mill lade location with other explanatory variables in the future.

## Supplementary Material

pgae251_Supplementary_Data

## Data Availability

All datasets and computer scripts necessary to reproduce the results in the study are provided in the public repository (Figshare, https://doi.org/10.6084/m9.figshare.c.6806952 or in its online supplementary material. **License information**: No claim to datasets from which data in this study were derived: OS data under ©Crown Copyright 2007, License number 100017572; OS data under ©Crown Copyright [and database right] (2021); data under ©The Canal & River Trust copyright [database right] (2015); results based upon Land Cover Map 2021 under ©UK CEH copyright (2022) and ©Environment Agency copyright [and database right] (2016); and data from the SEPA and HadUK-Grid under an Open Government License v3.0.

## Notes

a The third and fourth schools of thought are broadly represented by Robert Allen (30) and Andreas Malm (14). Allen argues that the main drivers were cheap coal and expensive labor that stimulated innovations in labor-saving machinery driven by steam engines, whereas Malm argues that the adoption of steam engines was to deskill labor, centralize production to better access urban workforces and thereby drive down production costs. In both cases profitability is increased for factory owners, but they weigh causal factors differently in their interpretations.
