# Supplementary material for: Limited waterpower contributed to rise of steam power in British “Cottonopolis”
Source: PNAS Nexus. 2024 Jul 16;3(7):pgae251. doi: 10.1093/pnasnexus/pgae251 (PMC11249955; doi:10.1093/pnasnexus/pgae251)
Supplement: pgae251_Supplementary_Data [file pgae251_supplementary_data.zip › WaterpowerIndustrialBritain_Supplemental_final_plain.pdf]

Supplementary Appendix

Limited waterpower contributed to rise of steam power in British 'Cottonopolis'

Tara N. Jonell, Peter Jones, Adam Lucas, and Simon Naylor

Corresponding author: tara.jonell@glasgow.ac.uk

The PDF file includes:

Supporting text
Figures S1 to S5
Supplemental References

Other supporting materials for this manuscript include the following:

Tables S1 to S6
Data S1

Supporting Text

Waterpower potential estimates

Historic Climate. The early 19th century was arguably one of the coldest and driest intervals over the last millennium (Fig. 2). Reconstructed global mean surface temperatures over the past 2,000 years show significant cold temperature anomalies after 1800 CE and before industrial-era warming (1). Paleoclimate reconstructions across the late 18th and mid-19th centuries (1770–1840 CE) indicate that reduced solar irradiance during the Dalton Solar Minimum (1790–1830 CE) (2, 3), enhanced volcanic activity (4, 5), and a cold-phased North Atlantic promoted colder- and drier-than-normal conditions throughout the British Isles and Europe (6-10). Specifically, the Old World Drought Atlas (8), built from European, African and Middle Eastern tree ring datasets from 890–2012 CE, notes a 1779–1827 CE drought period comparable to earlier outstanding medieval 'megadrought' events, with 1770–1840 CE ranked as one of the driest multi-decadal periods over the last millennium (10). A decreasing trend in storminess (i.e., gale-day frequency) (11) is documented across this period and flood-poor conditions were pervasive across the British Isles, NW France, and Germany (12-14) after 1790 CE and into the early 19th century. Early instrumental and meteorological observations together with weather diaries lend further support to drier conditions across England, Wales, Scotland, and Ireland (15-19), with recorded 'droughty' conditions in British historic news texts (20) and English canal records (17, 21). While many of the above studies agree on the presence of drier-than-average conditions across the early industrial-era British Isles, the absolute magnitude and duration of historic drought events remain controversial.

Here we utilize the first decade of systematic observations (1891–1901 CE) to best approximate early industrial-era moisture conditions. Only sparse rain gauge coverage and very few direct observations of temperature exist before systematic recording (22). Gridded precipitation datasets are available from 1862 CE (23) but potential evapotranspiration datasets calibrated by historic temperature observations exist from 1891 CE onward (24, 25). The period from 1850–1900 CE is the most reasonable surrogate period for pre-industrial-era climate, with only slight industrial-era warming (+0.05 ºC) observed by that time (26-28). We argue that the 1891–1901 CE decade of the meteorological time series may be especially appropriate as a surrogate for the cool and dry early 19th century, given that 1891–1901 CE is the first decade of the 'Long Drought', a two-decade period (1890–1910 CE) of broadly cooler and drier conditions than observed in the immediately preceding and following decades (20). The 1891–1901 CE interval further features reduced solar (2, 3) and enhanced volcanic activity similar to 1770–1840 CE phenomena, affecting decadal and centennial-scale climate variability across the North Atlantic (7, 11, 28, 29). Given evidence that 1770–1840 CE may have been drier than any of our moisture scenarios, it is possible that absolute waterpower availability remains overestimated in this study.

Runoff-weighted flow accumulation for each cell with >1 km^2 drainage area was used to calculate average discharges for four intervals: i) early historic (1891–1901 CE), ii) long-term historic (1891–1940 CE), iii) series average (1891–2015 CE), and iv) modern (1961–2015 CE). Respectively, these intervals broadly represent: a drier historic interval before significant modern warming; a long-term interval before significant modern warming; the series average; and a modern interval subject to significant modern warming.

Power Post-processing and Assumptions. Following pre-processing and calculation of waterpower following flow-routing techniques (Materials and methods), raw power estimates were aggregated every ~150 meters (three cell or pixel average) along river to minimize any remaining spikes in power data. Networks defined by the Ordnance Survey (OS) as lakes,

canal watercourses, and river reaches subject to tides (30-32) were excluded. Post-industrial-era barriers and obstructions on waterways were catalogued from open datasets in late 2022/early 2023 provided by the OS, Scottish Environmental Protection Agency, Environment Agency, English Canal and River Trust, Global Database of Dams from OpenStreetMap, Highland Council, and include data from the UK CEH (30), Improvement Service Scottish Local Government (32, 33), Future Hydropower Reservoirs and Dams Database (FHReD) (34), Global Georeferenced Database of Dams (GOODD) (35), the Global Reservoir and Dam (GRanD) v1.3 Database (36), and the Georeferenced global Dams and Reservoirs (GeoDAR) v1.0 dataset (37) (table S2). Falls created by barriers were masked from power calculations using 100-meter buffers (Fig. 3 and fig. S3).

Gross theoretical power potential, while informative, is of limited practical value since local landcover, hillslope, contributing drainage area, and human infrastructure affect placement, cost and capacity of both modern (38, 39) and historic waterpower sites. Therefore, we further adjust estimates by excluding land undesirable for mill siting: regions above 300 m asl and within broadly-classified moor- and peatland (40), following observations in this study (Materials and methods) and from literature (41). The 'Moorland Line', drawn-up in 1992 CE and updated in 2007 CE (42, 43), largely coincides with the region unoccupied by 90% of historic mills. Within England, it encloses land defined as predominantly semi-natural upland vegetation, with or without rock outcrops, and used primarily for rough grazing. Here we define land unsuitable for milling as land above 300 m asl and landcover classified as acid grassland, heather, heather grassland, fen, marsh, swamp, bog, mire, and salt marsh in the 10-meter UK CEH Land Cover Map (44, 45). We recognize that modern landcover likely underestimates the coverage in the past. After 1600 CE and especially from 1750–1800 CE, common land essential for grazing and foraging changed dramatically through enclosure, drainage and privatization (46, 47). Compared with only 3% registered common land today, the 17th century featured ~20–30% of common land in lowland areas, with areas exceeding 70% in NW England and NW Scotland (47). Theoretical and exploitable power potential maps are presented for Britain (Figs 1 and 3) and summarized by 1851-era historic counties (48-51) and for 112 delineated rivers basins (tables S3 and S4). Power density (kW per km^2) by county and basin are shown in Figure S4.

Our estimates cannot be considered absolute values for historic waterpower given the data gaps and critical assumptions above. Nonetheless, the new estimates presented here based on mean annual runoff and modest efficiencies offer robust relative waterpower estimates at a much higher spatial resolution than those previously available (52, 53). Site assessment and total technical hydropower calculations require several characterizations of flow variability and the power capacity of waterpower technology that cannot be easily assumed here. Ideally, the flow-duration curve based on several years of river gauge records should be used so that hydrological variability can be captured. However, such a task is challenging at the national-scale due to limited historic observations for monthly rainfall, let alone more frequent time-series for river gauging. It is impractical, within the present effort, to interpolate and analyze daily time-series for each channel pixel given the temporal resolution of historic rainfall and evapotranspiration datasets.

We assume that changes in land-use patterns did not significantly change evapotranspiration but may have affected where historic mills were sited (47). We further note that long- and short-term groundwater contributions, as well as water abstractions and additions, are deemed negligible. We surmise this would lead to waterpower underestimates for regions with strong groundwater contribution (e.g., SE England) and overestimates for highly industrialized modern urban areas (54); although neither are the focus of this study. Similarly, our estimates do not account for lake overspill which could lead to localized

underestimates in power. We further assume no additions to power through impoundment by mill pond or reservoir. If mill ponds were used, they typically abstracted no more than several hours to one day's worth of water before returning water downstream (55, 56).

Historic waterpower utilization

The 1838 Factory Return. The 1838 Return of Factories (57), also referred to as the 1838 Factory Return, is one of the earliest systematic reports of power and employment in textile mills across the UK. It is widely considered to be the first comprehensive statistical report for many 19th century textile mills (56, 58, 59). From textile firms, inspectors requested data relating to type of power used, the motive power employed by engine(s) or waterwheel(s), and the number, age and gender of persons employed in cotton, lint (flax), woolen, worsted, and silk mills. The report compiles inspector data for all counties at the time by individual administration areas, which largely coincided to either a single civil parish or group of parishes.

From the 1838 Factory Return, we use installed waterpower capacity as a generous proxy for waterpower in-use, or waterpower demand. Waterpower demand was compiled for the major cotton, worsted, wool, flax, and silk textile sectors, converted to SI units (1 hp = 745.69987 Watts). We note that reported power often represents the maximum working power capacity by engine(s) and/or waterwheel(s) rather than average day-to-day power use. Data were manually geolocated to the nearest reported civil administrative center at the scale of civil parishes before aggregation at the county- and basin level (Figs. 1-3; table S5). Cumulative water- and steam-power were spatially aggregated according to the boundaries of historic counties set by the 1851 Census of Great Britain. These are the earliest digitized historic county boundaries available for all nations. They can be considered representative for the period of interest and before major changes in county boundaries occurred in the late 19th century. It is noted that by 1851 the boundaries of English and Welsh counties had already undergone changes by elimination of outlier county portions by 1844 (48, 50, 51). We note that earlier summaries for power and mills by Ref. (59) utilized modern county boundaries (c. 1979), and so their aggregated power and mill numbers (see Cartographic Mill Database below) may differ slightly from ours.

Other Milling Activities and Water Transport. We note that watermills were not necessarily all working simultaneously in operation on a watercourse at any one time for a variety of reasons. Either due to waterpower requirements for milling activities, mill and waterwheel type, or water rights, mills often abstracted water sequentially down the watercourse through the accepted or legislated workday (41, 55, 59, 60). Therefore, it is important to note that historic waterpower estimates here show the full potential relative to the expected full usage of waterpower by all textile mills. While waterpower utilization may not have reached that shown in Figure 3, the magnitude of difference in utilization between the Mersey Basin relative to elsewhere in Britain indicates how highly worked rivers in that region had become. Significant care was required to manage water resources by numerous parties across the basin (17, 21, 61). Estimates here (Figs. 3, S4) establish an absolute waterpower usage limit for each basin, arguably checking further 19th century industrial growth (62-66). Lastly, while our datasets inform only on power utilization by textile mills focused on mechanized spinning and weaving, our data lend insight as to how much natural water remained available for canal transport and other industrial activities in the early to mid-19th century. The 1838 Factory Return (57) does not account for the full diversity of milling

activities encompassed by the textile industry nor the use of power in all mills. Power used for the pre-processing of material like waulking (fulling) of wool and carding of cotton, or for the finishing of products through bleaching, dyeing, and printing are not accounted for. And perhaps most critically, the 1838 Factory Return, and thus our estimates for utilization, do not account for other industrial milling activities such as smelting and forging, that required significant amounts of waterpower (41, 59, 67).

Note on Prior Waterpower Potential Calculations. The only basin indicating greater waterpower potential than predicted by earlier work (53) is the River Irwell (707 km^2) in the Mersey Basin, one of the most industrialized rivers producing textiles throughout the classical period of the Industrial Revolution. New historic waterpower potential estimates (13.3–21.4 MW) exceed earlier estimates by 133–232% (4 MW; see table S1). Such an apparent increase in potential, given that all other basins are only 29–44% of prior estimates, raises questions about the drainage previously considered for the 'River Irwell' by Ref. (53). Prior estimates for the River Spodden (6 MW)(53), a tributary of the River Irwell, exceeded earlier total estimates by Ref. (53) for the 'Irwell'. We suggest it likely that the 'Irwell' of Ref. (53) represents the Upper Irwell tributary (167 km^2) popularly considered in 1835 CE by Sir Edward Baines (62), rather than the entire River Irwell encompassing the Upper Irwell, the River Spodden, and several other notable tributaries considered here (table S4). Taking this into consideration, we find that new estimates for the Upper Irwell reach only 39–68% of earlier findings and are comparable to other basin data presented here.

Cartographic Mill Database: 'Mills of Britain, 1729–1914 v1'

To-date, no central register of early or post-industrial mills exists in Great Britain. Although it has often been claimed that 20,000 mills existed throughout Britain by the modern period (68), this number remains to be ratified. This present work is informed by several partial mill 'censuses' (41, 59) and built from several open-access databases to construct a georeferenced industrial-era mill database for Britain. Almost all mill locations (1729–1914 CE) were recorded from digitized maps, with select mills recorded from undigitized large-scale county maps held at the British Library.

The Roy Gazetteer. Data from 1747–1755 CE were derived from the collaborative National Library of Scotland and British Library Roy Gazetteer project (https://maps.nls.uk/roy/gazetteer/) that transcribed placenames from the Roy Military Survey of Scotland map sheets. Raw locations and mill types were derived by either mill symbol (ideographs) or text via toponymy (69, 70). Text in the Roy Gazetteer was queried for English and Scottish Gaelic terms for 'mill' and 'mills', allowing for several spellings and phonetic variations. This included but was not limited to such terms such as mill, *miln*, *muileann*, *mhuillin*, *vulen*, *vuilin*, *vulan*, *voulin*, *mulen*, *mulin*, and milton. Text including words with geographic or topographic context were excluded if they i) were a duplicate location near a textually labelled mill in the Roy Gazetteer, ii) had no association with milling at that location at any point in the interval considered here (c.1729–1914 CE), or iii) were found to exist at a location nontypical for British watermills (see Point Pattern Analysis of Historic Mill Locations) (69). Locations associated with, but not limited to, words such as -burn, -town, -ton, -mains, -millgen, -scar, -craig, -crag, -pond and -dam and their plurals were removed from the dataset if no association as indicated above was found. All mill locations were corrected for distortion during georeferencing of maps, and i) collocated to typical

geomorphic locations of mills (Materials and methods) or ii) co-located to antecedent mills mapped from pre-Ordnance Survey and/or Ordnance Survey maps (see below), when possible and deemed acceptable. Watermills were also identified through their graphical depiction with a water lade in the Roy Military Survey maps, or lastly, labelled as a plausible watermill when falling within a 275-meter buffer after (69) on either side of the delineated IHDTM river network (30) in QGIS. Mills falling outside of the buffer were labelled as mills using muscle-power (animal, human) or wind power, and were thus not considered in watermill point pattern analysis.

Pre-Ordnance Survey County Maps. Data from 1729–1836 CE are derived from pre-Ordnance Survey (OS) county maps held by the NLS, the British Library, the National Library of Wales, the Royal Geographical Society (with the Institute of British Geographers), the National Library of France, the Digital Archive at McMaster University Library, the Yale University Library, the David Rumsey Map Collection at the University of Stanford Library. Data were manually collected from maps surveyed and published between 1729–1836 CE in Scotland, England and Wales (71-209). County map surveys were conducted often at the one- to two-inch to the mile scale, and more rarely, up to four-inches to the mile. We aimed to include at least one map surveyed between 1729–1790 CE and one map surveyed after 1810 CE for each historic county. Data in this interval (1729–1836 CE) cover almost all British counties, including the counties of Cheshire, Lancashire and Yorkshire that are widely considered the most industrialized regions in Britain in terms of textile milling. In pre-Ordnance Survey county maps, watermills were identified most often by ideograph; with waterwheel symbols the most common graphic indicating watermills (59, 70). Locations, mill types, and power usage, if indicated, were manually recorded from indicated symbols and text on map sheets. Plausible watermill sites with ambiguous symbology or text (i.e., buildings or circles) indicative of the primary motive power (water, wind, animal, steam) were filtered as above, using a 275-meter buffer adapted after Ref. (69) on either side of the delineated IHDTM river network (30) in QGIS. Mills falling outside this buffer were excluded from point pattern analysis.

Ordnance Survey Maps and the Scottish Water Mills Project. Data from 1843–1914 CE Ordnance Survey maps were filtered from the 'Scottish Water Mills Project' hosted by the National Library of Scotland (https://maps.nls.uk/projects/mills/), a 'census' of 9,795 georeferenced and classified mills, mill dams, and lades. This project was created by Iara Nave Calton at the University of Glasgow and funded in part by the UK Arts and Humanities Research Council. The 'Scottish Water Mills Project' was built from the GB1900 Gazetteer, a crowdsourced project transcribing 2.2 million place names from the 2nd edition six-inch to-the-mile maps of England, Scotland, and Wales (1888–1913 CE; https://data.nls.uk/data/map-spatial-data/gb1900/), and data from the first comprehensive map surveys of Scotland in the 1st edition six-inch to-the-mile (1843–1882 CE) and 2nd edition 25-inch to-the-mile (1892–1914 CE). In that project, data were collated with archaeological, architectural, and aerial survey data from Historic Environment Scotland's National Record of the Historic Environment ("Canmore") database. In this study, we focus solely on the textile industries of cotton, lint (flax), wool, worsted, and silk. We therefore filtered out all mills associated with these terms: rags, barley, block, flint, slate, saw, paper, threshing, horizontal, madder, barytes, corn, farina, flour, grain, charcoal, crushing, distillery, bone, snuff, saw, bark, barrel, smelt, gunpowder, iron, dyewood, rice, wood, brewery, brick, calendar, clay, chemical, engine, fertilizer, glue, hair, net, shovel, soap, sugar, tannery, tile, timber, turning, and works. We

further removed all windmills, sluices, dams, reservoirs, and ponds from the dataset for our analyses. We note that datasets from 1729–1836 CE currently do not allow for detailed filtering like the 'Scottish Water Mills Project' dataset.

Ordnance Survey Maps and English and Welsh watermills. Data from 1888–1914 CE were derived from the GB1900 Gazetteer that transcribed placenames from the 2nd edition six-inch to-the-mile maps of England and Wales. Text was queried for English and Welsh terms for 'mill' and 'mills', allowing for several spellings and phonetic variations. This included but was not limited to such terms such as mill, *miln*, *myle*, *myln*, *melin*, *melindwr*, *felin*, *melinau*, *felinau*, *argae*, and pandy. Text serving as qualitative descriptors, e.g., mill bank, mill ford, *melindref*, *nant y felin*, for infrastructure or geographic features, were removed. Text words were also excluded if they i) were a duplicate location with English and Welsh labels, or ii) were found to exist at a location nontypical for British watermills (see above). The same filters as above were also used to isolate mills associated with the five main textile industries in this study.

On the Accuracy of Early Maps. Early maps, like all maps, are interpretations informed by evidence but inclusive of error and subject to bias. Records of mills from large-scale county surveys are therefore a product of the original conditions and time of their surveyance, the quality of their identification and representation by surveyors, the preservation of engraved maps over time, and their later interpretation from symbology and/or text (210, 211). A thorough consideration of mill numbers in county maps before 1800 CE suggests no more than ±15–20% error in the most rural areas of Scotland and Wales, with ±5–10% average error across pre-OS county maps (59). However, omission of mills in growing urban settings becomes apparent in early 19th century county maps before more systematic OS mapping, in part due to limited space for graphical representation. Generally omission affects water-powered mill numbers less than steam-powered mills in highly urban settings, and large textile mills less than small grain mills in rural settings (70, 212). Repeated counts of mills produce no more than ±5% error (this study relative to Ref. (59)). In the database we note, where possible, both the years of survey and publication, because pre-OS surveys often occurred more than a decade before publication (210). In most cases, mill numbers from cartographic maps offer a robust but reliable minimum at the same order of magnitude of in-situ mills captured through other archival evidence such as statistical accounts and inspection reports.

Recirculating Engines in the Mersey Basin

We utilize the most comprehensive, public database of early steam engines, the Early Steam Engine Database (https://coalpitheath.org.uk/engines/) and Ref. (213) to assess our findings relative to the chronology of early steam engine installations in the 18th century. From the database (2023 version), 346 steam engines are identified to have been deployed in cotton mills between 1780 and 1802 CE. The vast majority were in England, with only 23 of those recorded in the database deployed in Scotland. Of that total, 94 are not recorded as having any specific function, 189 are generically recorded as used in 'textile manufactories' (all of which were cotton mills), and 61 others are specifically recorded as being used for recirculation of water. The three remaining engines are recorded as being used for 'backup', winding and to drive machinery. With regard to the recirculating engines, 16 are recorded as Savery engines and 33 are Newcomen engines. However, an additional six of the 33

Newcomen engines recorded are not listed as being used for recirculation, but could only have been used for that purpose, bringing the total involved for water recirculation to 67 engines. Of the recirculating engines returning water to cotton mills, only 64 of these are precisely located (213), with more than one-third of these (24 engines) adopted in the upland tributaries of the Upper Irwell, Roch, Croal and Tame tributaries and in lowland Manchester city. Precise locational data can be acquired from the Early Steam Engine Database, with recirculating steam engine numbers summarized by county for the Mersey Basin in Supplementary Table s6.

Descriptions of Waterpower in Early Industrial Britain

We have compiled published descriptions of milling and waterpower in Britain from 18–19th century historical contemporaries and excerpts from the scholarly work of mill historians, economic historians, archaeologists, and historians of technology. However, it is important to note that while the passages below give valuable context to the early Industrial Revolution and water scarcity from historical contemporaries, none are from textile manufacturers or mill owners themselves.

All emphases in bold are added, with notes in brackets provided for clarity. Many of the historical texts are available online through open digital libraries, such as the Internet Archive.

Opinions from 19th century historical contemporaries on power and textile milling

1. Oliver Evans was an influential inventor and engineer credited with publishing one of the most used texts on milling in the 19th century, *The Young Mill-Wright and Miller's Guide* (214) in 1795 CE. Text from *The Young Steam Engineer's Guide* (215), a re-issue of his earlier text *The Abortion of the Young Steam Engineer's Guide (216)* published in 1805 CE, is often referenced with regards to mills, power and the Industrial Revolution:

> "**Water-falls are not at our command in all places**, and are liable to be obstructed by frost, drought, and many other accidents. Wind is inconstant and unsteady: animal power, expensive, tedious in the operation, and unprofitable, as well as subject to innumerable accidents. **On neither of these can we rely with certainty. But steam at once presents us with a faithful servant, at command in all places, in all seasons; whose power is unlimited**; for whom no task is too great nor yet too small; quick as lightning in operation; **docile as the elephant led by a silken thread, ready, at our command, to rend asunder the strongest works made by the art of man**."

2. The following excerpts are from the *Rees Cyclopædia* (https://www.biodiversitylibrary.org/bibliography/59683), edited by Abraham Rees and written by approximately 100 contributors between 1802 and 1820 CE. Below are some excerpts from entries for 'MILL'(217), 'WATER'(218) and 'STEAM' (219), with some that are self-contradictory. John Farey Jr., a 19th century mechanical engineer of some repute and author of *A Treatise on the Steam Engine* in 1827 CE (220), is listed as a main contributor to the overall topics of 'WATER' and 'STEAM'.

In WATER, *On the Distribution of the different Falls of Water in* Rivers, page 97 (218):

> “**Upon most rivers in this country all the falls of water are fully occupied, and at every mill there is a weir, which pens up the water as high as the mill above can suffer it to stand without inconvenience**. Each miller is anxious to obtain the greatest possible fall, and he can at any time augment the fall, by raising the surface of his weir; **but as this may produce an inconvenience to the mill above, in preventing the water from running freely away from its wheel, it is a constant source of dispute and litigation**. A mill may be subjected to tail-water by the occurrence of so many circumstances, that it is frequently very difficult to know where to seek the best remedy, whether the miller ought to raise his wheel higher and diminish his own fall, or insist upon a diminution of his neighbour’s below him by lowering his weir.”

[Note: This is followed by a detailed discussion about tail-water problems and issues between millers on this topic.]

Despite the passage above by Farey et al., it is stated there were abundant falls not used under WATER, in *Gardening*, page 134 (218):

> “However, waterfalls may either, it is supposed, be imitated directly, by being copied from nature, or indirectly, by the introduction of weirs for the use of water-mills, as already hinted …
>
> … The nature of waterfalls for the purpose of driving machinery are, is observed, generally pretty well understood … **But it is remarked that it is to be regretted that so few who have rivers take advantage of it, and so many make cascades equally formal and unnatural, without any real use …**”

Anonymous commentary about the nuisance of watermills due to their regulation of water, under the topic of ‘MILL’, whose volume was first published in 1812 CE, page 539 (217):

> “**Water-mills have long been great nuisances to agriculture**, by preventing the use of the streams on which they stand, in many cases, in irrigating and flooding the adjoining lands, by which much improvement is kept back, that would otherwise take place. They are also injurious by obstructing and damming up the water in numerous instances, so as to render it stagnant on the ground above. **Wind and steam may, however, be applied as the moving powers of mills without producing any such effects, and are, of course, the most proper powers to be employed**.”

[Note: The same sentiment is reiterated in WATER-*Mill*, in *Rural Economy*, p. 148-149 (218)]

3. Sir Edward Baines, a 19th century journalist and later Member of Parliament, is lauded for his work on the history of cotton manufacturing in Lancashire. He is one of the most oft-quoted historical contemporaries on the topic. Baines wrote in 1835 CE (62) on p. 220-221 about the limitations of waterpower and its apparent check upon the cotton industry. Emphasis in italics in the original text:

> “**Amazing as in the progress which had taken place in the cotton manufacture prior to 1790, it would soon have found a check upon its further extension, if a power more efficient than water**

> **had not been discovered to move the machinery. The building of mills in Lancashire must have ceased, when all the available fall of the streams had been appropriated**. The manufacture might indeed have spread to other counties, as it has done to some extent; but it could not have flourished in any district where coal as well as water was not to be found; and **the diffusion of mills over a wide space would have been unfavourable to the division of labour, the perfection of machine-making, and the cheapness of conveyance**.
>
> At this period a power was happily discovered, of almost universal application and unlimited extent, adapted to every locality where fuel was cheap, and available to both make machines and to work them, both to produce goods, and to convey them by land and water. This power was the *steam-engine*, which, though not an invention of that age, was first made of great and extensive utility by the genius of James Watt."

4. Sir Robert Kane was a promoter of waterpower for industrial use. Kane was a 19th century chemist of international standing. His 1845 CE book on the various natural resources of Ireland, *The Industrial Resources of Ireland* (52) comments on the disjunction between the availability of waterpower and the wide adoption of steam engines in the British Isles. He is arguably the first individual who published early calculations for theoretical waterpower potential for industrial use in Britain.

On page 67 (52), Kane comments on steam power:

> "It is only of late years that in this country the steam-engine has nearly superseded the use of air and water as a prime-mover. Until recently steam has been auxiliary to water; it is now the principal source of power, and waterfalls are of comparatively small value, except in certain districts. **So long as water was depended upon, the mills of Great Britain and Ireland were necessarily circumscribed in their operations and diminutive in size; they have now become so colossal, that they require steam-engines of much greater power than the largest water-wheels, and there appears to exist no limit to the magnitude and importance to which they may yet attain**."

Kane succinctly states why steam power had become so widely adopted, page 108 (52):

> "Why then is steam so much used? In the first place, waterpower is available only in certain localities, where other more influential circumstances may forbid the introduction of manufactures; and secondly, **the influence of the cost of power is generally so small in mechanical industry, that the question of savings in regard to it is swallowed up in more important questions**."

Commenting on the ubiquity of coal in England and lack of interest in early machines similar to the early water turbine, page 90 (52):

> "The water pressure engine is a machine, but little known in this country [Ireland]. In fact, borrowing as we do our mechanical ideas

from **England, a country, generally speaking, so rich in fuel as to render the economy of waterpower unimportant, water engines do not fix the attention of mechanists as they deserve**."

In an excerpt below with some text omitted for brevity, Kane outlines the major difficulties associated with the natural irregularity of waterpower. On p. 96-97 (52):

> "**The worst feature of waterpower is that its production does not depend upon the will of the person who employs it**…[T]he amount of water flowing along any given river cannot be estimated for any time in advance, and hence the manufacturer has open to him but one or other of two courses, each of which is beset with disadvantages ... In the first place having estimated the smallest quantity of water that is available in summer, he may proportion his machinery, and work to that amount of power. But by doing so, he sees by him through the greater portion of the year, power many times as great as that which he economizes… The powers which could execute in a day that which occupies his factory for a week, are running to waste… The alternative course by which the machinery is constructed of power sufficient for the employment of the average quantity of water is accompanied by not less evils. In fact, in the dry season, industry is arrested; there is not enough water to work the mill… orders remaining unexecuted, contracts unfulfilled, may entail the loss of the most serious kind upon whose capital is invested in such works…
>
> **I believe that the disadvantages to which waterpower is liable are fully expressed in the above statement. Now, I do not hesitate to say that they are all capable of being removed. If we give to the conditions of waterpower the same care that is bestowed upon the circumstances of steam power, those disadvantages disappear, and we obtain from water, during the year, a steadiness of supply, and a regularity in work, that leaves nothing to be desired**."

[Note: This is followed by lengthy discussion about how water reservoirs could negate most of the issues outline above. Kane then presents an examination of the cost of power at Shaws Water Works in Greenock (Scotland) by engineer and consultant Robert Thom, and a similar reservoir in Ireland to underline the waterpower potential and savings.]

Commentary on waterpower resources in the Mersey Basin

1. <u>River Irwell</u>

Below [bolded text] is perhaps one of the most quoted examples of mill crowding and lack of adequate fall for waterpower in British Industrial Revolution literature. It is taken from Baines' 1835 CE book, *History of the Cotton Manufacture* on p. 85-86 (62). When commenting on the River Irwell and its tributaries, Baines writes:

> "…In the early part of their course, these streams and streamlets furnish water-power adequate to turn many hundred mills:* [footnote 1] they afford the element of water, indispensable for

scouring, bleaching, printing, dyeing, and other processes of manufacture…

*Footnote: **On the river Irwell, from the first mill near Bacup, to Prestolee, near Bolton, there is about 900 feet of fall available for mills, 800 of which is occupied**. **On this river** [the Irwell] **and its branches, it is computed that there are no less than three hundred mills.** A project is in course of execution to increase the water-power of the district, already so great and so much concentrated, and to equalize the force of the stream, by forming eighteen reservoirs on the hills, to be filled in times of flood, and to yield their suppliers in the drought of summer. These reservoirs, according to the plan, would cover 270 acres of ground, and contain 241,360,000 cubic feet of water, which would give power equal to 6,600 horses. The cost is estimated at £59,000. One reservoir has been completed, another is in course of formation, so it is probably that the whole design will be carried into effect."

[Note: The remaining reservoir scheme was never completed. Few scholars have examined Baines' comment on the planned reservoir scheme nor impact on waterpower in industry.]

2. <u>River Irk and Medlock (tributaries of River Irwell)</u>

Edwin Butterworth, a 19th century journalist and historian, is perhaps best known for his work on the local history of Oldham, one of the most lucrative textile manufacturing centers in England. Butterworth worked closely with Baines, as is reflected through similar phrasing to Baines' text in the following excerpt in *Historical Sketches of Oldham…* (221) on p. 134:

"…It is thus evident that, including Lees hall lower mill, eight new cotton mills were established from 1788 to 1791, and, deducting one concern which appears to have ceased about 1783, the number of cotton manufactories in the township of Oldham in 1791 was eighteen. **All these mills were moved by water or horsepower. If no superior power had been discovered to move the machinery, the cotton manufacture would have soon found a check upon its further extension**, but happily the genius of James Watt rendered the steam engine of almost universal application, adapted to every locality where fuel was cheap, and available as well to make as to work machines, and capable of both producing and conveying goods by land and water."

The following excerpts from Butterworth (221) describe the mixed power usage at Oldham, necessitated by its location, on p. 130 and 232, respectively. Historical Oldham was centered on an interfluve between the Rivers Irk and Medlock, two tributaries of the River Irwell:

"All these new manufactories were moved by horse power, with one partial exception, Lower Sheepwashes mill, which was worked by both horse and water power."

"**Its elevated situation and the absence of any rivers in the neighbourhood, rendered it dependent on distant places for its supply.**"

Following the quotes from Butterworth (221) above, Hills wrote in *Power in the Industrial Revolution* on p. 91 (222) that:

> "By 1788 there were about twenty-five mills in the whole of Oldham parish, eleven of them in Oldham itself while by 1791 there were eighteen in Oldham alone. **Because the best water sites had been taken** [in Oldham], most of these mills were driven by horses [cites Butterworth, 1856] but there was a very high casualty rate owing to the numbers of improvements and the increases in size of the spinning machines which made old machines uneconomic."

[Note: It is not clear what is meant by 'best water sites' given the general location of Oldham and the mixed power usage at mill sites.]

3. River Croal (tributary of River Irwell)

Richard Hills was a leading 20–21st century historian of technology and power in the Industrial Revolution, who discussed motive power in relation to physical location, the needs of manufacturers, and the technology available at the time. Hills, in *Power in the Industrial Revolution* on p. 93 (222), notes the inadequacy of rivers at large centers for cotton manufacturing that were unable to handle escalation in waterpower usage:

> "The best sites on the larger rivers could produce considerably more power than the early steam engines, and water was free while coal cost money…In the earliest days, those areas which did not have adequate water resources were at a serious disadvantage. The main cotton textile areas were Lancashire with the fustian and calico trade, and Nottingham and Derby with hosiery. **Although the cotton industry had been established at Bolton for very many years, Aiken** [in 1795 on p. 262 (223)] **noted that shortage of water in this area was affecting the type of yarn produced**:
>
> > 'Since the opposition of the populate to the use of machines for shortening labour has been quelled by convincing them of their utility, spinning factories have been erected **throughout all the surrounding country, especially where water is plentiful**. **The streams near Bolton are too near their sources to furnish the water that large works require**; there are a few therefore, in its neighbourhood of the larger kind, though several of the smaller … The want of water in this district is made up by the ingenious invention of the machines called mules.'"

[Note 1: Hills utilizes the descriptions of John Aikin, an 18–19th century medical doctor, who meticulously described his travels around Manchester: https://wellcomecollection.org/works/c88vcjba/items.]

Hills (222) comments on Aikin (223) and Butterworth (221) on page 95:

> "Aikin mentions many waterpower mills in his *Description…Round Manchester*, and the passages already quoted from Butterworth about the horse mills **show how many small mills were set up on streams which seem barely adequate to supply any power at all** …"

At Bolton [along the River Croal, a tributary of the River Irwell], John Kanefsky comments on the general correlation between adoption of steam power and a lack of water on page 516 in *The diffusion of power technology in British industry 1760-1870* (59). Kanefsky is one of the foremost 20-21st century historians on steam engines, their records, and their historic adoption in the British Isles.

> "Being one of the principal cotton spinning and finishing areas of Lancashire and because **water power was in short supply Bolton saw considerable early use of steam engines and at least 18 engines were erected there in the eighteenth century**."

4. River Tame

Aikin (223) describing the River Tame and the town of Ashton-under-Lyne:

> "**It is well-supplied with water, except about two months in the summer, when the inhabitants are obliged to fetch their soft water in carts from the Tame.** This river abounds with trout. It is also of the highest utility to the machinery of the woolen and cotton factories of the neighbourhood; it being reckoned that **within the space of ten miles from Ashton there are near 100 mills upon this stream and its tributary branches**."

[Note: The reference by Aikin to ten miles around Ashton is a considerable area at 314 sq. miles or 814 km^2.]

5. River Goyt

Hills on page 97 notes the influence of the physical setting and geology of Stockport, another major center of cotton production in Greater Manchester:

> "A glance at any map of the Manchester area will show how the industrial towns and villages follow the river valleys and how the mills, situated at the falls in the rivers, helped to determine the position of the early industrial settlements. **Stockport** [River Goyt-Tame confluence] **is a particularly good example, for here the river cuts across the line of the buried Permian ridge and the river gradient is steepened**. The silk industry used waterpower here soon after 1736, and by 1800 **the gorge was choked by a long line of mills. 'Undoubtedly Stockport's status as the prototype of the Lancastrian mill-town was a direct outcome of its abundant water-power resources, by far the finest of any site within the low land'**."

[Note: Hills cites *Manchester and Its Region: A survey for The British Association*, p. 13 (224)]

By 1795, Aikin notes use of steam engines in Stockport, page 446 (223):

> "The cotton trade at Stockport is now so considerable, that besides a large number of cotton spinning shops, there are twenty-three large cotton factories, four of them worked by steam engines."

On the landscape, water resources, and mill crowding in the Mersey Basin (broadly)

While there are various legal and social issues that could arise from mill crowding, as well as later environmental effects as outlined by Downward and Skinner (225) and Walter and Merritts (226) in England and the NE United States, crowding at any one location does not necessarily show competition or pressure on water resources. Crowding could also result from: i) singular ownership of multiple mills at one location; ii) shared infrastructure like lades, weirs and millponds between multiple parties (although less common); and iii) the size and geometry of the river itself. Fluvial knickpoints, including waterfalls, on moderately-sized rivers provided ideal places to site watermills due to a combination of discharge and fall, among other favorable factors in terms of cost (see Refs (69, 227).

1. von Tunzelmann, in *Steam Power and British Industrialization to 1860*, quotes the number of mills (i.e., mill crowding) across several river basins in England to argue there was pressure on waterpower resources, on p. 137-138 (58):

> "**Several authors seem to consider that until the last decade of the eighteenth century there was little pressure at all on water resources in those areas that were to achieve industrial predominance in the years to come** (e.g., Hills, p. 102 [(222)]; Chapman, p. 2 [(228)]). **Thereafter the take-up was rapid.** Unwin described a race for water-power at Stockport [River Goyt] in 1790-1 (Unwin, p. 123-128 [(229)]). Lancastrian spinners and manufacturers moved to Flintshire or Furness in search of water (Chapman, p. 8-16 [(228)]; Tupling, p. 204-205 [(230)]). Samuel Crompton mentioned as many as 44 mills on the upper reaches of the River Aire at the time of his census in 1811. In Gloucestershire, 24 mills were concentrated on less than 5 miles of the River Frome (Chapman, p. 19, [(228)]). Baines wrote of the congestion of water wheels on the River Irwell by the 1830s—800 out of 900 feet of fall being fully occupied (Baines, p. 86 [(62)])."

[Note: These examples demonstrate the fallacious basis of using 'mill crowding' as a proxy for waterpower without further context. The River Aire catchment is ~1,000 km^2, the River Frome is ~470 km^2, and the section of the River Irwell that Baines (62) considered is 167 km^2.]

2. From *Manchester and Its Region*: *A survey prepared for The British Association*, p. 6-8 (224) describing fluvial knickpoints:

> "Glaciation had other economic consequences in the upland valleys, for it largely governed the availability of water-power. **All the master-streams, the Goyt, Tame, Irwell, and Etherow, are in general, too well-graded to be good power-sources. But, at intervals, all have been forced from their pre-glacial courses by drift-masses** which plug the old valleys. Here, the deflected stream has been forced to cut a new course around the drift-barrier, and thus is has become incised into the gritstone flanks of its old valleys in the narrow slot of a diversion gorge. Within the gorges the stream flows quickly and turbulently; between them it meanders over a broad, alluvial valley-floor. **These short reaches were by far the best power-sites, and they were quickly taken up by the early river-powered cotton mills.**"

3. Industrial archaeologist Owen Ashmore in *The Industrial Archaeology of Lancashire* (231) comments on the concentration of industry along particular river valleys and at specific locations. From p. 14-15:

> "The steep slopes and the narrow valleys of the high land which runs along the eastern boundary from the Ribble to the Mersey basin, extending westward in the Rossendale hills, provided encouraging conditions for the early growth of textile manufacture. **A fairly heavy rainfall** [relative to other areas of England], **well distributed throughout the year, helped to produce both the humid atmosphere traditionally associated with the cotton industry and the clear, swift-flowing streams which supplied power for the early mills and lime-free water for manufacturing processes …**"

Ashmore (231), on p. 40 and 43:

> "The use of water-power determined the location of most of the early mills along the rivers and the tributaries which run down from the Pennine and Rossendale moors—**the Irwell, Irk, Tame, Roch, Calder, and Darwent.** Many were built in remote sites away from any town and difficult of access. The pressure on water-power resources is illustrated by the way in which, in some of the river valleys, carding and spinning mills were built one above the other at quite short intervals…"

[Note: These 'short intervals' are likely the km-scale fluvial knickpoints with exceptional waterpower, as noted above.]

Hills (222) discusses how the required power at a water powered mill site changed over time, and how this related to the growth in power needs of mills, page 193:

> "The size of each mill would have been determined not only by the power available but by other factors such as supply of the raw cotton, the availability of labour and the size of business which could be properly administered with the existing facilities of postal services, wholesalers and so on. Presumably the first cotton mills were supplied by pack-horse, so that **transport costs may have tended to limit the size of the first water-driven mills** which had to be situated in the countryside. As river, canal and road transport facilities improved, the cost of carriage must have fallen and the size of loads increased. Both of these would have meant that larger units of production could become possible. **Therefore the mill owner who had a mill which had been established for a few years might find himself with a unit which could be profitably expanded but his original source of power could not be increased.**"

Commentary on waterpower resources in the Midlands

1. River Tame of Birmingham

Kanefsky (59) comments on the early adoption of steam-engines in the Midlands. First, on River Tame mills, page 229:

> "The area was not especially well endowed with water resources, both the gradients and the flows of the streams being only moderate. The area therefore switched to steam power at a fairly early date as neither the number of sites nor the output at each was adequate for the demands of the metal-working and other industries in the area."

And about Birmingham and the West Midlands, pages 407-8:

> "The Black Country and Birmingham are somewhat unusual in that their **water power potential was very limited and economic development quite rapid, so that there was little opportunity to use water power as a basis for operation**. Even those mills which did exist suffered from drought problems and this was one of the reasons why Matthew Boulton was interested in Watt's inventions (cites Pelham (232)). Water power therefore tended to be replaced by the more reliable and more powerful steam engine at an early date in the West Midlands."

2. <u>River Leen (Nottingham)</u>

Stanley Chapman, a notable British historian of the Industrial Revolution, comments on mill crowding on underfit rivers in 'The Cost of Power in the Industrial Revolution in Britain: the case of the textile industry', p. 5 (56):

> "But the real context for the new development was provided by the pressure on the diminutive resources of the River Leen and other sites in the locality of Nottingham. By 1784 there were at least 17 mills on this rivulet,* a sluggish stream only a dozen miles long, and with a fall of only 156 ft over the entire length (OS 6-inch map)."

[*Note: Chapman also wrote that the Spodden (of the River Irwell) was small and unfit, although the trunk river falls 235 meters (~770 feet) over 12 km (~7.45 miles).]

Commentary on waterpower in the 19th century

4. Kanefsky comments on the Rivers Don, with its tributaries of the Sheaf, Rivelin, Porter Brook and Loxley in Yorkshire on page 230 (59):

   > "Though Sheffield was similar to the West Midlands in that few new water power sites were available it did not experience a rapid transition to steam power as the numerous powerful streams of the area could supply much of the requirements of the metalworking industries until after 1850 ... as more efficient use was made of the potential by increasing the head or installing new wheels."

5. Jenkins and Ponting, in *The British Wool Textile Industry 1700-1914*, p. 120-122 (233) outline the continued use of waterpower in many regions across Britain:

   > '**The use of water for power survived for many decades, particularly in the industry away from the main centres,** and can be traced without difficulty from the Factory Returns until 1871. But

from the 1874 Factory Returns onwards details of power were no longer recorded. In 1850 in England and Wales 35 per cent of the nominal horsepower recorded was produced by water, but this figure no doubt overstates the importance of water as the nominal horsepower measurement was becoming increasingly unrealistic, especially for steam engines. But in that year, according to the recorded figures, water was producing the majority of power for the woollen industry in the counties of **Gloucester, Somerset, Cumberland, Oxford, and a number of others. In Devon, Westmorland, Shropshire, Herefordshire, and Dorset, waterwheels were still providing all the power** for the local industry and in Wales steam power had made very little progress. In Scotland, 65 per cent of nominal horsepower was generated by water ... **[W]ith the exception of the counties of Lancashire and Wiltshire, water power still predominated in other woollen manufacturing areas.** Only a few worsted manufacturing counties were, however, so dependent on water and the industry in a number of counties including **Norfolk** [East Anglia] **and Leicester** [Midlands] **used little or no water power.**

… Water power continued to be used in most areas of the British wool textile industry until the twentieth century. **But with the exception of the very rural parts of Wales, Scotland, and the Border Counties of England, few mills relied entirely on water. Some of those had been initially located, decades earlier, close to a good supply of water for power, continued to use that water but supplemented the power it provided with a steam engine**, which also enabled the mill to continue production in times of excess or dearth of water."

6. Kanefsky on abundant waterpower in the Lake District (59) of NW England, page 230:

"In contrast to the West Midlands the number of watermills in the Lake Counties doubled in the first half of the nineteenth century, from 350 or so to over 700. The average power of these mills must also have increased significantly as many of them were large iron, textile, wood turning, and gunpowder mills using tens (and in the case of gunpower hundreds) of horsepower. The water power capacity of the region therefore more than doubled, from perhaps 4,000 to 10,000 horsepower. **The reason for this were largely topographical, the area being abundantly supplied with hitherto untapped water power potential which could be used for mills of 30, 40, or more horsepower**."

7. Chapman on the availability and cost of sites on page 2 (56):

"**The rent of water power sites might be thought to be a critical variant, but there is evidence to suggest there were plenty of cheap sites on the market in the later decades of the eighteenth century** … In the Pennines, declining industries like lead and iron

led to the offer of further sites, and increasing specialisation in pastoral farming in the upland regions may have produced a similar result (Chapman, *Early Factory Masters*, 1967). There were plenty of country landlords wanting to augment the value of their estates by introducing or extending manufactures (Chapman, 1967). **Most important of all, perhaps, the total power demands of cotton, the most active sector of the textile industry, were still modest; the total import of cotton in 1796 could have been carded and spun with 5,000 h.p** (This figure must be compared with the rich resources of water power in Lancashire alone. 'A typical minor stream draining to the Manchester embayment, the Spodden, yielded 428 h.p. at 18 sites spread over five miles of its course.' [citing Rodgers, 1960, p. 138 (234)]"

[Note: There were likely not as many old mining sites useful for textile mills. Based on observations of mill locations in this study, no more than 1% of all mills were located above 300 meters in upland regions. And often those very few that were above 300 meters were mills for pumping groundwater from the mine and related mining activities, i.e., crushing materials and washing coal and mineral ores. Even if these sites became unoccupied, most were not suitable for non-mining industry (i.e., textile mills) that did not pump groundwater. Overland flow (runoff) may not have supplied much power at all in such upland rivers with small drainage areas.]

8. Chapman on power on p. 19-20 (56), with some text omitted for brevity:

> " … water was capable of competing with steam power throughout the long period (1780-1850) of transition from the domestic to the factory system in cotton textiles where sites near large towns could be developed. **The problem was to find sites that could generate sufficient power for the growing scale of the industry, but reserves of water power were lacking in the appropriate areas especially because the Pennine streams were parcelled out between numerous proprietors … The limited and diminishing power available on these sites must be contrasted with rapid strides in the optimum scale of textile mills in the later 1830s, and the 1840s**. Again most of the evidence is available for cotton, but as cotton was, technically speaking, the leading sector of the textile industries, it is of greatest interest. Comparison of the larger (2,000 spindle) Arkwright prototype mill of 1795 with the type of mule spinning and power loom weaving plant recommended by Montgomery in 1836, suggests that **while productivity had trebled over the half century and fixed capital requirements had increased slightly, the power requirement was still only 20 h.p.; but this was the turning point …**
>
> … The perfection of Roberts' 'self-acting' mule in the 1830s encouraged a growth in size … The best sites, like Holywell, might generate 100 h.p. [74 kW], but 10 or 20 h.p. [7–15 kW] was a typical limit, and there was little chance of meeting the power requirements of the new cotton mills on such slender natural resources."

Commentary by economic historians on energy, power, and the Industrial Revolution

Below are selected quotes from 20-21st-century economic historians who discuss motive power and energy limitations in relation to the Industrial Revolution and modern era.

1. Commenting on the take-up of the steam engine and the use of waterpower in the 19th century in *The Growth of English Industry and Commerce* (235), p. 626-627, William Cunningham notes:

> "Its full effect was only gradually felt, and **water continued to be economically the better agent during the first quarter of the nineteenth century**; but eventually as a consequence of Watt's invention, water-falls became of less value."

2. Similarly, Arthur Redford, writing in 1931 in *The Economic History of England, 1760-1860* (236), p. 111, similarly argues:

> "There was during the earlier nineteenth century a notable expansion in most of the manufacturing industries of England ... The expansion was especially remarkable in the cotton industry ... **Water power was still more important than steam power**, even in the most progressive of manufactures; **it was not until the second quarter of the century that the steam engine could be said to have ousted its rival**, and in most other industries the change came later."

3. Phyllis Deane (63) on the irregularity and supposedly limited potential of wood, waterpower, and windpower in the mid-late 18th century, in *The First Industrial Revolution*, on p. 129-130 (63):

> "**The most crucial and general of the bottlenecks limiting the expansion of the British economy on the eve of the British industrial revolution (that is in the middle of the 18th Century) were two: they were of wood and the shortage of power** … The only forms of power available to the pre-industrial economy were muscle-power, water-power, and wind-power. None of these was capable of development to form which would support a modern industrial economy. **The watermill and the windmill had been available for centuries and a good deal of ingenuity had been put into their design. But they were subject to two inescapable limitations—they were erratic and unpredictable** in that they depended on weather conditions (and there is nothing less predictable than the British weather), and it was necessary for the power they generated to be used on the spot … Wood was a dwindling resource with a strictly limited future as a construction material in an industrial context. **Water-power and wind-power were only partially under the control of their operators and had a very limited potential,** The power of the average windmill or

> water-wheel was in the region of 5-10 h.p. and in their most elaborate and expensive forms they seemed unable to generate more than 30 h.p.

[Note: Deane's comment about waterwheels seeming 'unable to generate more than 30 h.p. is simply incorrect, as the nine waterwheels at New Lanark generated 400 h.p., one of which was capable of generating 70 h.p. (59), p. 142, while the world's largest waterwheel, the Laxey wheel on the Isle of Man was capable of generating 185–200 h.p.. See also the previous quote from Kanefsky (59), p. 230, and accounts regarding Stanley, Deanston and Catrine mills in Scotland, among many others.]

4. In David Landes' *The Unbound Prometheus: Technological Change and Industrial Development in Western Europe From 1750 to the Present* (237), p. 99, he comments that if Britain had had better water resources and less coal resources, it would have continued to use waterpower for much longer:

> "**In the eighteenth century and the first decades of the nineteenth, the water wheel accounted for the greater, though a diminishing, share of the power used by British industry**; and **there is no doubt that, had Britain been better endowed by nature with hydraulic energy,** or had she been poorer in coal, **the dominance of the wheel would have continued much longer than it did**. This was the case in the United States, where the great coal deposits lay in what were at first the relatively inaccessible lands west of the Appalachians and where the eastern slopes of the same range offered superb sites for the erection of water driven mills. The same was true of comparable areas in Europe, the whole Alpine region, for example—Dauphiné, Switzerland, Baden, Bavaria, northern Italy."

5. Sir Anthony Wrigley on the lack of fall and waterpower [likely with reference to the cotton industry only] in 'The Process of Modernization and the Industrial Revolution in England', p. 249 (64):

> "The power requirements of the new textile machines [1770s water frame and spinning jenny] were so modest that the steam engine was no more than a useful alternative to a waterfall for many years. The overshot wheel provided all the power that was needed to drive them. **But there came a time when greater power was needed and unused waterfalls with a sufficient head were few and remote**. Even in the textile industry, and still more in industrial production at large, it is reasonable to assume that the factory would not have become the predominant unit of production without the new technology of coal, steam, and iron."

6. From *Continuity, Chance and Change*, Wrigley comments on the limited potential and irregularity of water- and windpower relative to modern energy requirements, p. 75 (238):

> "For most industrial and agricultural processes human muscle could be supplemented only by animal muscle, and, great though the value of such assistance was, it was necessarily limited and generated the same competing pressures for access to a scarce resource common to all animate life in an organic economy. Other sources of mechanical power, principally the windmill and the waterwheel, though not subject to the same problems, were not capable of transforming the overall power situation. Wind energy was intermittent and apt to be most abundant on sites that were least convenient in other respects. **Water power was also subject to seasonal variations and interruption according to the vagaries of precipitation and the effects of freezing weather, and was subject to rising marginal costs of provision since the better sites were naturally developed first, leaving smaller or less conveniently situated falls for later exploitation.** Quite apart from these disadvantages, and not withstanding their great value for particular applications**, neither wind nor water power was available on a scale sufficient to hold out any hope of radically transforming the prospects for output per head and hence of living standards**."

7. Wilkinson, in *Poverty and Progress: an ecological model of economic development*, on the lack of waterpower sites engendering steam engine adoption, p. 120-121 (239):

> "**The use of water power was limited by the number of streams with suitable sites for mills …** While enough sites were available [referring to an earlier time period], water wheels remained the easiest and most economical way of obtaining a rotary motion for powering mills … **It was not until the late eighteenth century, when the new cotton mills began to add to the demand for rotary power and good mill sites were no longer available**, that Boulton and Watt made the first steam-engine harnessed to produce a rotary motion ... The substitution at a later date of steam power for the rotary power of mills and horse-whims was a response to growth in demand for rotary power at a time when the supply from traditional sources was relatively fixed."

[Note: Wilkinson's attribution of the motivation for the invention of rotary steam engines to a 'relatively fixed supply' of power from 'traditional sources' is not supported by any evidence].

8. Pomeranz on waterpower and modern energy requirements in *The Great Divergence: China, Europe, and the making of the modern world economy*, p. 59-61 (240):

> "Coal was central to earlier views of the Industrial Revolution. Only cotton, iron, steel, railways got comparable attention, and except for cotton, these other main sectors depended on coal. But more recently, coal has often been deemphasized. **People have noted, for instance, that more early factories were powered by water than by coal** and that most of England's coal was used for the

unglamourous and not particularly innovative tasks of home heating and cooking … Nonetheless, at least a partial return to the earlier emphasis on coal seems warranted, both for Wrigley's reasons and for others. **Water may for a time have powered more mills than coal, but it was geographically restricted, nonportable, and often seasonally unreliable…[W]aterpower, no matter how much the wheels were improved, simply did not have the same potential to provide energy inputs that would significantly outpace a rapidly growing population for decades to come or to permit chemistry to substitute for land."**

9. Joel Mokyr on Wrigley (241) in *The British Industrial Revolution: an economic perspective*, p. 32 (242):

   "Britain had coal and iron, but coal and iron were traded commodities … Above all it should be recalled that much of the Industrial Revolution depended on cotton and that raw cotton was entirely imported … Trade, it should always be remembered, liberates nations from the arbitrary tyranny of resource location … In other words, it is possible to accept Wrigley's (1987) view that substituting coal for wood was an important part of the economic transformation of Britain, without attributing undue significance to the geographical accident of the presence of coal in Britain. Coal had substitutes; as fuel, coal-poor nations like the Netherlands and Ireland relied on peat, while the mountainous areas of Europe relied on water power. Such substitutions involved costs, of course, but the examples of Switzerland and New England prove that water power could provide an adequate energy base for a mechanized industry."

10. Paul Warde (243) on energy shortages, sites and waterpower in *Energy Consumption in England & Wales, 1560-2000*, p. 15 (243):

    "Phyllis Deane [(63)] hypothesised that developments in manufacturing technology can be linked directly to **a need to overcome an 'energy shortage' deriving from both wood shortage and industrial [plant] saturating all of the sites that could reasonably be used for water power in the most intensely industrialized regions**. More recent studies however have tended to emphasize **the persistent importance of waterpower in manufacturing until the second half of the 19th century, while Gordon** [(53)] **has argued that England remained far from exploiting all the possibilities of water power**."

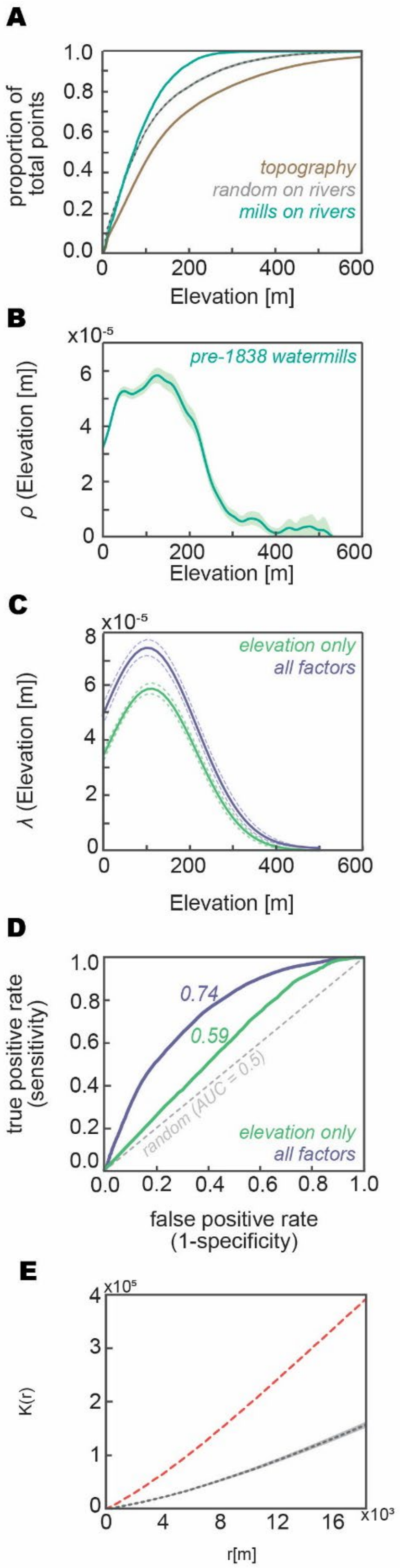
A
1.0
0.8
0.6
0.4
0.2
0.0
proportion of total points
topography
random on rivers
mills on rivers
0
200
400
600
Elevation [m]
B
x10-5
pre-1838 watermills
ρ (Elevation [m])
6
4
2
0
0
200
400
600
Elevation [m]
C
x10-5
8
6
4
2
0
λ (Elevation [m])
elevation only
all factors
0
200
400
600
Elevation [m]
D
1.0
0.8
0.6
0.4
0.2
0.0
true positive rate (sensitivity)
0.74
0.59
random (AUC = 0.5)
elevation only
all factors
0.0
0.2
0.4
0.6
0.8
1.0
false positive rate (1-specificity)
E
x10⁵
4
3
2
1
0
K(r)
0
4
8
12
16
x10³
r[m]

Figure S1. **Exploratory point pattern analysis of historical mill dependence on elevation.** **(A)**, Empirical cumulative density function (eCDF) of all mill location (n=15,570 sites) elevations relative to a spatially random homogeneous Poisson point distribution (n=100,000 trials); **(B)**, Nonparametric dependence estimation of pre-1838 watermill location (n=11,849 sites) intensity by elevation, with confidence interval shaded in green; **(C)**, Loglinear quadratic models simulating watermill density based on elevation only (green line) and another model fitted to elevation, mean upstream accumulation (≈ drainage area) and mean hillslope gradient within 1000 meters of the river channel (purple line); **(D)**, Measure of aggregated model performance indicated by the receiver-operating characteristic curves (ROC) for models fitted to i) elevation only (green) and ii) elevation, mean upstream drainage area, and hillslope gradient within 1000 meters of the river channel (purple line). Dashed lines indicate confidence interval. The area under the ROC curve (AUC) is 0.7422. Values closer to one indicate perfect discrimination, where 0.8> AUC >0.7 indicate good to excellent discrimination by the model. **(E)**, Empirical inhomogeneous K-function indicating stronger clustering by real watermill locations (red dashed line) than simulated by prior model (black dashed line) including confidence intervals (gray shaded region).

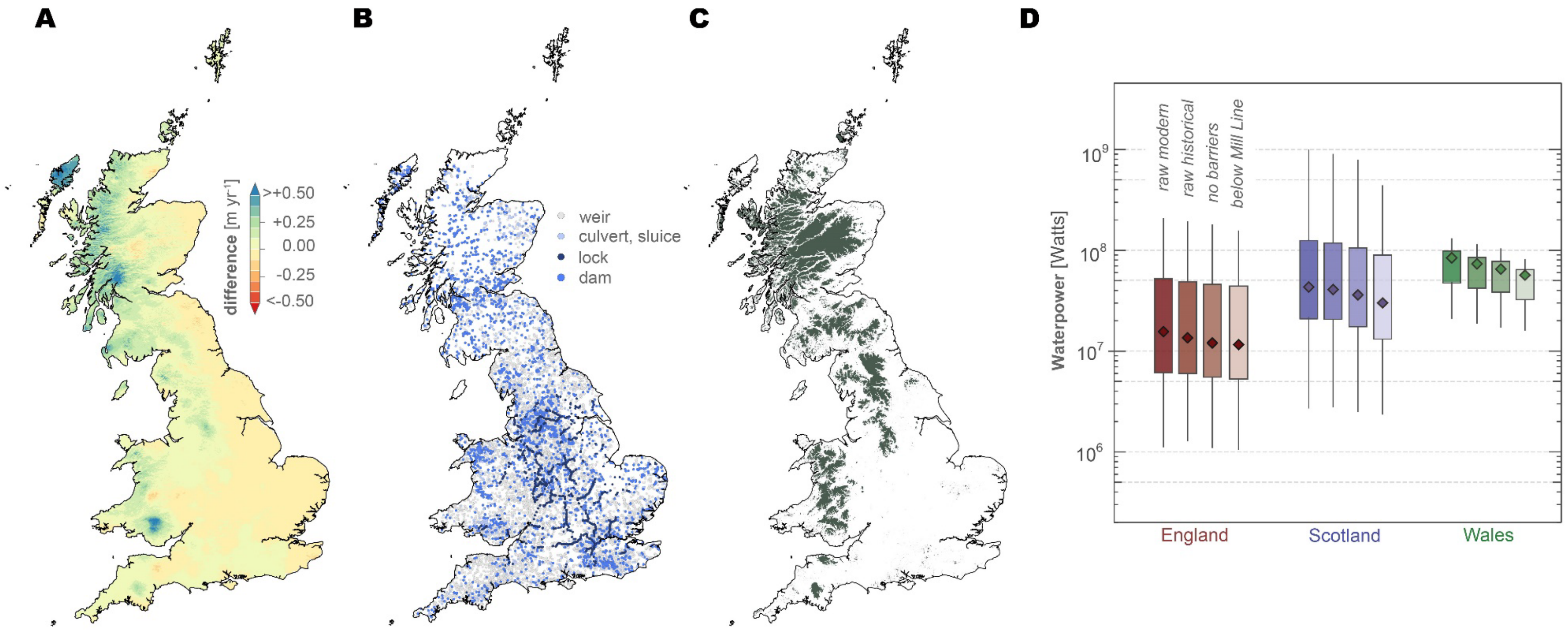

Figure S2. Moisture, waterway barriers, and upland vegetation corrections to waterpower potential. (A), Absolute difference between modern (1961–2015 CE) and early industrial (1891–1901 CE) moisture (P-PET) intervals. **(B)**, Catalogued post-industrial-era waterway barriers and obstructions (Table S1; data S1). **(C)**, Elevation and landcover (dark green polygons) not preferrable for milling based on historic mill site locations. Includes land above 300 m asl and landcover classified as acid grassland, heather, heather grassland, fen, marsh, swamp, bog, mire, and salt marsh. **(D)**, Average waterpower by British historical county for England, Scotland and Wales when corrected for historical moisture, waterway barriers, and for land below the Mill Line and outside moor- and peatland vegetation. Diamonds indicate mean values.

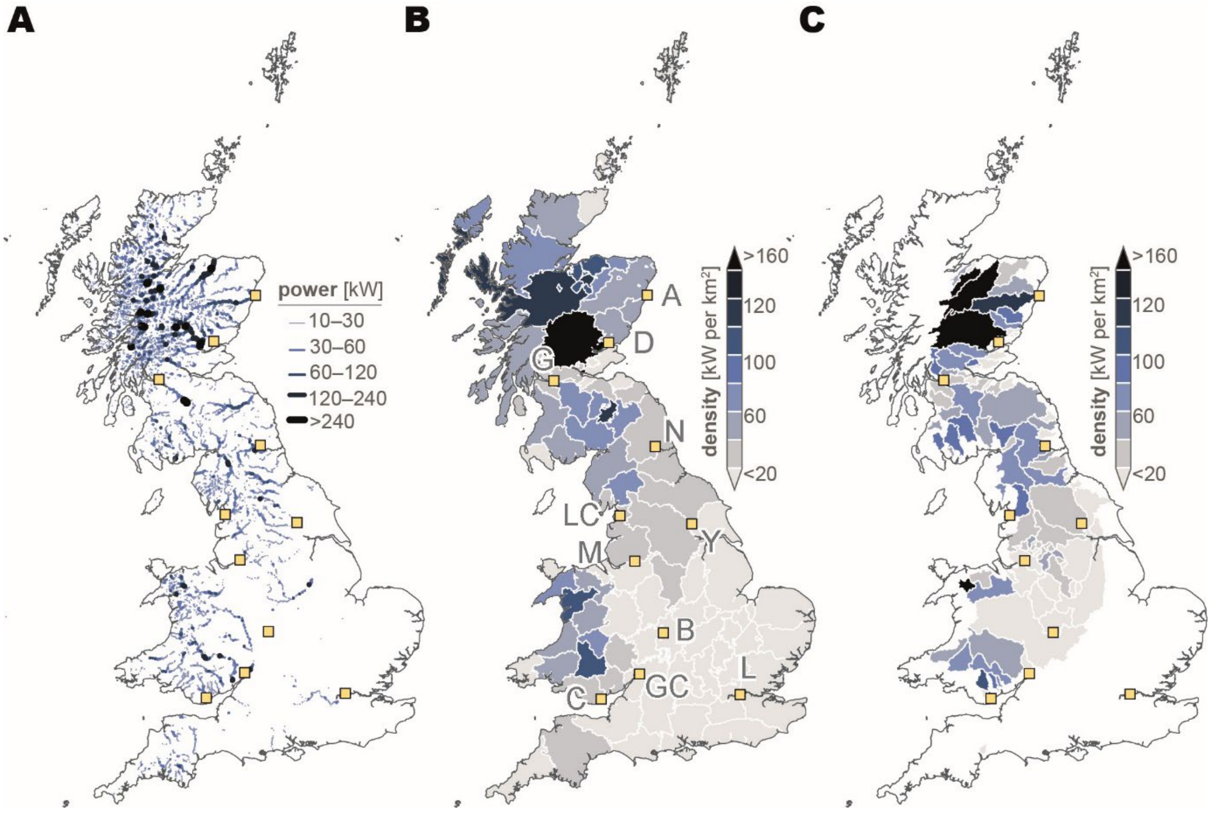

Figure S3. Historic waterpower potential for the late 18th-early 19th century (1770–1840 CE). (A), Raw theoretical waterpower potential. **(B)**, Historical waterpower density (kW per km^2) by historical county, corrected for post-industrial-era barriers and unexploitable land beyond the Mill Line. **(C)**, Historical waterpower density (kW per km^2) by river basin, corrected for post-industrial-era barriers and unexploitable land beyond the Mill Line. Major city centers as yellow squares after Figure 1.

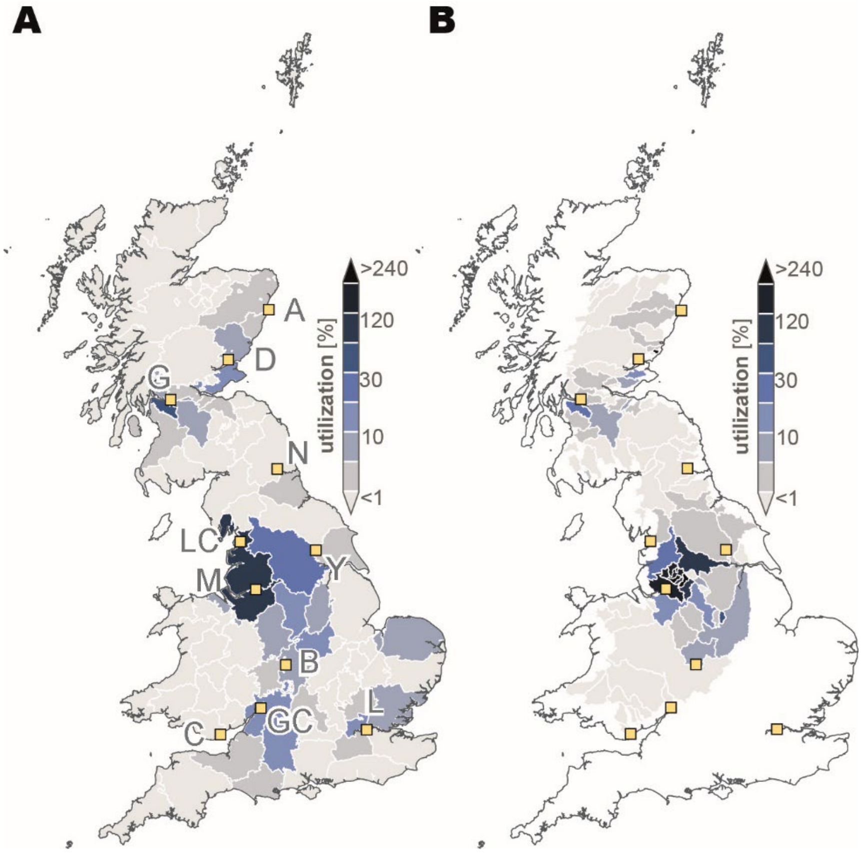

Figure S4. Utilization of historic waterpower potential following all steam and waterpower demand in 1838 Factory Return. (A) Utilization of natural waterpower by historical county. (**B**) Utilization of natural waterpower by river basin. Efficiency at 40%. Major city centers as yellow squares after Figure 1.

A

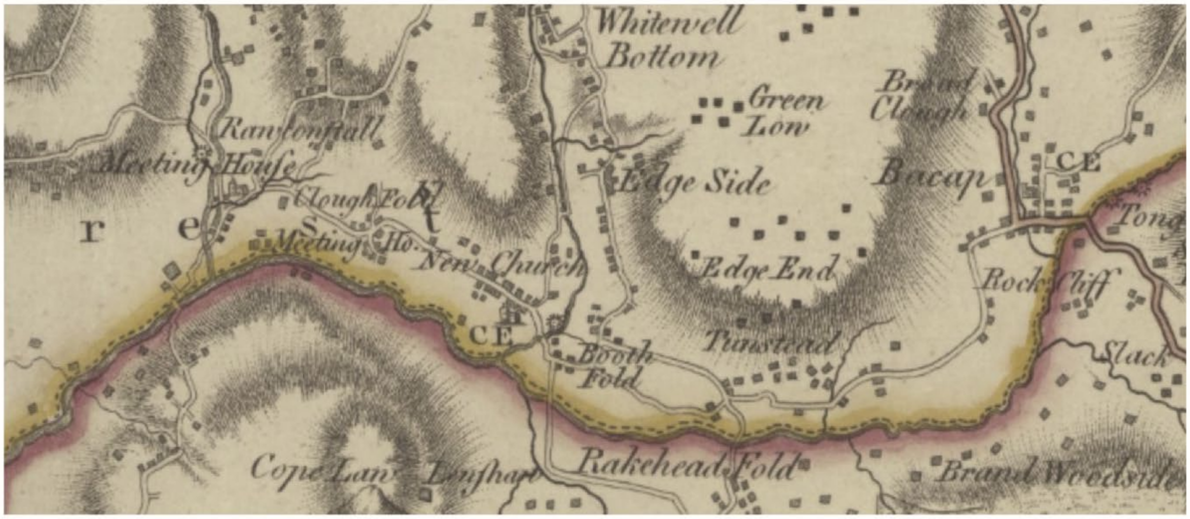
Whitewell
Bottom
Green
Low
Broad
Clough
Rawtonstall
Meeting House
Clough Fold
Edge Side
Bacap
CE
Meeting Ho.
New Church
Edge End
Rock Cliff
CE
Booth
Fold
Tunstead
Slack
Rakehead Fold
Brand Woodside

B

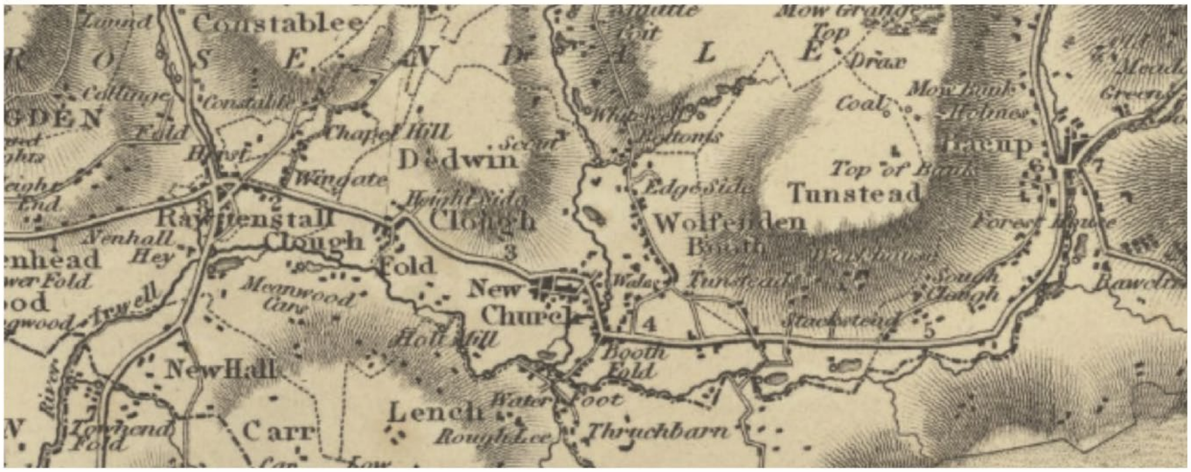
Constablee
Constable
Chapel Hill
Dedwin
Wingate
Tunstead
Rawtenstall
Clough
Edgeside
Wolfenden
Booth
Fold
New
Church
Booth
Fold
NewHall
Lench
Water Foot
Rough Lee
Thruchbarn
Carr
Irwell
Forest House

C

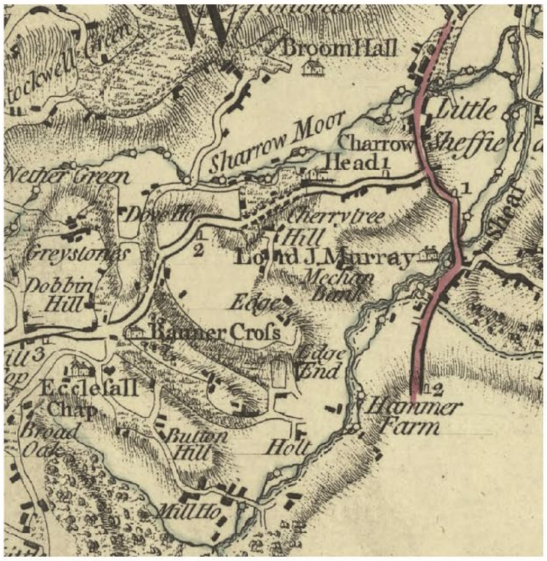
BroomHall
Stockwell Green
Little
Sheffield
Sharrow Moor
Charrow
Head
Nether Green
Cherrytree
Hill
Greystones
Lord J.Murray
Dobbin
Hill
Edge
Banner Crofs
Edge
End
Ecclesall
Chap
Broad
Oak
Button
Hill
Holt
Hammer
Farm
Mill Ho.

E

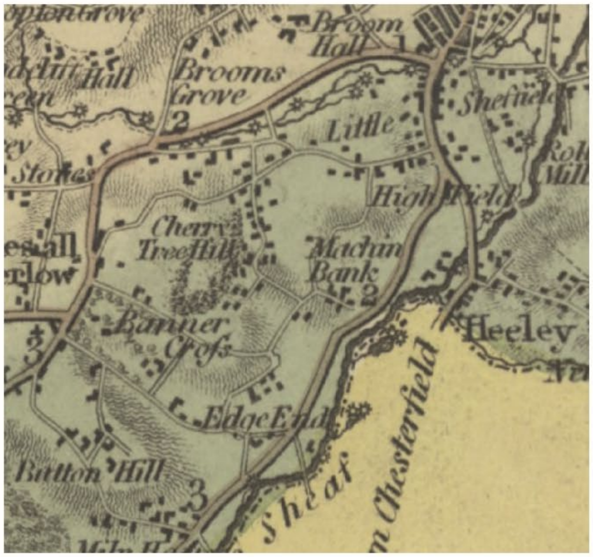
Broom
Hall
Brooms
Grove
Little
High Field
Cherry
Tree Hill
Machin
Bank
Banner
Crofs
Heeley
Edge End
Button Hill
Sheaf

Figure S5. Eighteenth- and nineteenth century county maps indicating watermills, lades, and millponds. (**A**) Yates' 1786 CE map of Lancashire (115) near Bacup on the Upper Irwell, the uppermost portion of the River Irwell of interest to Baines (62) (https://maps.nls.uk/view/220113073#zoom=6&lat=3642&lon=5965&layers=BT). Map survey likely completed before 1780 CE (59). Color indicates administration boundaries denoted by publisher, partly along the River Irwell; (**B**) Greenwood's 1818 CE map (138) of Lancashire at the same location (https://maps.nls.uk/view/220113091#zoom=7&lat=2089&lon=7022&layers=BT); (**C**) Jefferys' 1771 CE map of South Yorkshire (85) near Sheffield on the River Sheaf (https://digitalarchive.mcmaster.ca/islandora/object/macrepo%3A80965). Surveyed from Surveyed 1769–1770 CE; (**D**) Greenwood's 1817–1828 CE survey of South Yorkshire (182) at the same location (https://maps.nls.uk/view/220113187#zoom=7&lat=1472&lon=2212&layers=BT). Watermills represented by waterwheel symbols connected by lines (lades) to millponds and rivers. Images for A, B, and D provided by the National Library of Scotland under CC-BY and C by the Digital Archive at McMaster Library by CC BY-NC 2.5 CA. In-text images available only at low resolution; please use hyperlinks for high-resolution images.

Table S1. Comparison of waterpower potential from select river basins to prior estimates (held in figshare at https://doi.org/10.6084/m9.figshare.c.6806952).

Table S2. Catalogue of post-Industrial waterway barriers and obstructions (held in figshare at https://doi.org/10.6084/m9.figshare.c.6806952).

Table S3. Summary of historical waterpower potential and utilization by historic county (held in figshare at https://doi.org/10.6084/m9.figshare.c.6806952).

Table S4. Summary of historical waterpower potential and utilization by select river basin (held in figshare at https://doi.org/10.6084/m9.figshare.c.6806952).

Table S5. Summary of 1838 Factory Return by historic county (held in figshare at https://doi.org/10.6084/m9.figshare.c.6806952).

Table S6. Early recirculating steam engines adopted by cotton mills in the Mersey Basin (held in figshare at https://doi.org/10.6084/m9.figshare.c.6806952).

Data S1. Historic waterpower of Britain for GIS (held in figshare at https://doi.org/10.6084/m9.figshare.c.6806952).

References

1. Neukom, R *et al.* 2019. Consistent multidecadal variability in global temperature reconstructions and simulations over the Common Era. *Nature Geoscience*. 12(8), 643-649.
2. Usoskin, I, Gallet, Y, Lopes, F, Kovaltsov, G, Hulot, G. 2016. Solar activity during the Holocene: the Hallstatt cycle and its consequence for grand minima and maxima. *Astronomy & Astrophysics*. 587A150.
3. Lean, JL. 2018. Estimating solar irradiance since 850 CE. *Earth and Space Science*. 5(4), 133-149.
4. Gao, C, Robock, A, Ammann, C. 2008. Volcanic forcing of climate over the past 1500 years: An improved ice core-based index for climate models. *Journal of Geophysical Research: Atmospheres*. 113(D23).
5. Dawson, AG, Kirkbride, MP, Cole, H. 2021. Atmospheric effects in Scotland of the AD 1783–84 Laki eruption in Iceland. *The Holocene*. 31(5), 830-843.
6. Trouet, V *et al.* 2009. Persistent positive North Atlantic oscillation mode dominated the Medieval Climate Anomaly. *Science*. 324(5923), 78-80.
7. Mann, ME *et al.* 2009. Global Signatures and Dynamical Origins of the Little Ice Age and Medieval Climate Anomaly. *Science*. 326(5957), 1256-1260.
8. Cook, ER *et al.* 2015. Old World megadroughts and pluvials during the Common Era. *Science advances*. 1(10), e1500561.
9. Baker, A, Hellstrom, J, Kelly, BF, Mariethoz, G, Trouet, V. 2015. A composite annual-resolution stalagmite record of North Atlantic climate over the last three millennia. *Sci Rep*. 510307.
10. Ionita, M, Dima, M, Nagavciuc, V, Scholz, P, Lohmann, G. 2021. Past megadroughts in central Europe were longer, more severe and less warm than modern droughts. *Communications Earth & Environment*. 2(1).
11. Dawson, AG, Hickey, K, McKenna, J, Foster, IDL. 1997. A 200-year record of gale frequency, Edinburgh, Scotland: possible links with high-magnitude volcanic eruptions. *The Holocene*. 7(3), 337-341.
12. McEwen, LJ. 2006. Flood seasonality and generating conditions in the Tay catchment, Scotland from 1200 to present. *Area*. 38(1), 47-64.
13. Macdonald, N, Sangster, H. 2017. High-magnitude flooding across Britain since AD 1750. *Hydrology and Earth System Sciences*. 21(3), 1631-1650.
14. Bloschl, G *et al.* 2020. Current European flood-rich period exceptional compared with past 500 years. *Nature*. 583(7817), 560-566.
15. Smith, K. 1995. Precipitation over Scotland, 1757-1992: Some aspects of temporal variability. *International Journal of Climatology*. 15543-556.
16. Cole, GA, Marsh, TJ. 2006. The impact of climate change on severe droughts: Major droughts in England and Wales from 1800 and evidence of impact. Environment Agency, p 54.
17. Harvey-Fishenden, A, Macdonald, N, Bowen, JP. 2019. Dry weather fears of Britain's early 'industrial' canal network. *Regional Environmental Change*. 19(8), 2325-2337.
18. Murphy, C *et al.* 2020. Multi-century trends to wetter winters and drier summers in the England and Wales precipitation series explained by observational and sampling bias in early records. *Int J Climatol*. 40(1), 610-619.

19. O'Connor, P, Murphy, C, Matthews, T, Wilby, RL. 2022. Historical droughts in Irish catchments 1767–2016. *International Journal of Climatology*. 42(11), 5442-5466.
20. Dayrell, C *et al.* 2022. Representation of Drought Events in the United Kingdom: Contrasting 200 years of News Texts and Rainfall Records. *Frontiers in Environmental Science*. 10.
21. Harvey-Fishenden, A, Macdonald, N. 2021. The development of early reservoirs to supply water to arterial canals in England and Wales. *Landscape History*. 42(2), 79-98.
22. Pedgley, DE. 2002. *A short history of the British Rainfall Organization*.Royal Meteorological Society. p. 19.
23. Hollis, D, McCarthy, M, Kendon, M, Legg, T, Simpson, I. 2019. HadUK-Grid—A new UK dataset of gridded climate observations. *Geoscience Data Journal*. 6(2), 151-159.
24. [dataset] Tanguy, M, Prudhomme, C, Smith, K, Hannaford, J, Historic Gridded Potential Evapotranspiration (PET) based on temperature-based equation McGuinness-Bordne calibrated for the UK (1891-2015). NERC Environmental Information Data Centre. https://doi.org/10.5285/17b9c4f7-1c30-4b6f-b2fe-f7780159939c.
25. Tanguy, M, Prudhomme, C, Smith, K, Hannaford, J. 2018. Historical gridded reconstruction of potential evapotranspiration for the UK. *Earth System Science Data*. 10(2), 951-968.
26. Abram, NJ *et al.* 2016. Early onset of industrial-era warming across the oceans and continents. *Nature*. 536(7617), 411-418.
27. Hegerl, GC *et al.* 2019. Causes of climate change over the historical record. *Environmental Research Letters*. 14(12), 123006.
28. Hawkins, E *et al.* 2020. Observed emergence of the climate change signal: from the familiar to the unknown. *Geophysical Research Letters*. 47(6), e2019GL086259.
29. Hernandez, A *et al.* 2020. A 2,000-year Bayesian NAO reconstruction from the Iberian Peninsula. *Sci Rep*. 10(1), 14961.
30. [dataset] Moore, RV, Morris, DG, Flavin, RW, Sub-set of UK digital 1:50,000 scale river centreline network. UK Centre for Ecology and Hydrology.
31. Messager, ML, Lehner, B, Grill, G, Nedeva, I, Schmitt, O. 2016. Estimating the volume and age of water stored in global lakes using a geo-statistical approach. *Nature Communications*. 7(1), 13603.
32. [dataset] Renewable Energy Sites - Scotland. EDINA Digimap Ordnance Survey Service. https://data.spatialhub.scot/dataset/renewable_energy_sites-is.
33. [dataset] Culverts - Scotland. EDINA Digimap Ordnance Survey Service. https://data.spatialhub.scot/dataset/culverts-is.
34. Zarfl, C, Lumsdon, AE, Berlekamp, J, Tydecks, L, Tockner, K. 2015. A global boom in hydropower dam construction. *Aquatic Sciences*. 77(1), 161-170.
35. [dataset] Mulligan, M, van Soesbergen, A, Sáenz, L, GOODD, a global dataset of more than 38,000 georeferenced dams. https://doi.org/10.6084/m9.figshare.c.4648214.v1. Deposited 2020/01/21.
36. Lehner, B *et al.* 2011. High-resolution mapping of the world's reservoirs and dams for sustainable river-flow management. *Frontiers in Ecology and the Environment*. 9(9), 494-502.
37. [dataset] Wang, J *et al.*, GeoDAR: georeferenced global dams and reservoirs dataset for bridging attributes and geolocations. Copernicus Publications. https://essd.copernicus.org/articles/14/1869/2022/.

38. Environment Agency. 2009. *Good practice guidelines to the environment agency hydropower handbook: The environmental assessment of proposed low head hydropower developments*. Bristol: Environment Agency, p. 45.
39. Gernaat, DEHJ, Bogaart, PW, Vuuren, DPv, Biemans, H, Niessink, R. 2017. High-resolution assessment of global technical and economic hydropower potential. *Nature Energy*. 2(10), 821-828.
40. [dataset] Morton, RD, Marston, CG, O'Neil, AW, Rowland, CS, Land Cover Map 2020 (land parcels, GB). NERC EDS Environmental Information Data Centre.
41. Shaw, J. 1984. *Water Power in Scotland, 1550-1870*. Edinburgh: John Donald. p. 606.
42. Department for Environment, Food, and Rural Affairs Committee. 2011."Written Evidence" in *Environment, Food and Rural Affairs Committee Third Report: Farming in the Uplands*. Parliament of the United Kingdom (HC W 9 Feb 2011). https://publications.parliament.uk/pa/cm201011/cmselect/cmenvfru/556/55604.htm
43. Natural England. 2012. "Mapping the status of upland peat using aerial photographs" by Penny Anderson Associates Ltd. for *Natural England Commissioned Report NECR089*. Exeter: Natural England, p 72.
44. Jackson, DK. 2000. "Guidance on the interpretation of the Biodiversity Broad Habitat Classification (terrestrial and freshwater types): Definitions and the relationship with other Habitat classifications" in *Joint Nature Conservation Committee Report*. Peterborough: Joint Nature Conservation Committee, p 307.
45. [dataset] Morton, RD, Marston, CG, O'Neil, AW, Rowland, CS, Land Cover Map 2020 (land parcels, GB). NERC EDS Environmental Information Data Centre.
46. Winchester, AJL. 2000. Dividing Lines in a Moorland Landscape: Territorial Boundaries in Upland England. *Landscapes*. 1(2), 16-34.
47. Winchester, AJL. 2022. *Common Land in Britain: A History from the Middle Ages to the Present Day*. Woodbridge: Boydell Press. p. 328.
48. [dataset] Kain, RJP, Oliver, RR, Historic parishes of England and Wales: an electronic map of boundaries before 1850 with a gazetteer and metadata. UK Data Service.
49. [dataset] Southall, HR, Burton, N, GIS of the ancient parishes of England and Wales, 1500-1850. UK Data Archive.
50. [dataset] Satchell, M, Shaw-Taylor, L, Wrigley, EA, Kitson, PK, Newton, GH, 1851 England and Wales ancient counties. UK Data Archive.
51. [dataset] Satchell, M *et al.*, 1851 Scotland ancient counties. UK Data Archive.
52. Kane, R. 1845. *The Industrial Resources of Ireland*. ed. 2nd. Dublin: Hodges and Smith.
53. Gordon, R. 1983. Cost and use of water power during industrialization in New England and Great Britain: A geological interpretation. *The Eonomic History Review*. 36240-259.
54. Coxon, G *et al.* 2020. CAMELS-GB: hydrometeorological time series and landscape attributes for 671 catchments in Great Britain. *Earth System Science Data*. 12(4), 2459-2483.
55. Tann, J. 1965. Some problems of water power: A study of mill siting in Gloucestershire. *Transactions of the Bristol and Gloucestershire Archaeological Society*. 8453-77.
56. Chapman, SD. 1971. The cost of power in the Industrial Revolution in Britain: the case of the textile industry. *Midland History*. I1–23.
57. Parliamentary Paper XLII, House of Commons Factories Inquiry Commission. 1839 *Return of all the mills and factories specifying...the number of persons employed in*

cotton, woollen, worsted, flax and silk factories in the United Kingdom. London: House of Commons.
58. von Tunzelmann, GN. 1978. *Steam power and British industrialization to 1860*. Oxford: Oxford University Press. p. 356.
59. Kanefsky, JW. 1979. The diffusion of power technology in British industry. 1760-1870. University of Exeter [Ph.D. Thesis], p 583.
60. Musson, AE. 1976. Industrial motive power in the United Kingdom, 1800–70. *The Economic History Review*. 29(3), 415-439.
61. Sheail, J. 1988. River regulation in the United Kingdom: An historical perspective. *Regulated Rivers: Research & Management*. 2: 2221-232.
62. Baines, E. 1835. *History of the Cotton Manufacture in Great Britain*. London: Cambridge University Press. p. 220.
63. Deane, P. 1965. *The First Industrial Revolution*. ed. 2nd. Cambridge: Cambridge University Press. p. 332.
64. Wrigley, EA. 1972. The process of modernization and the industrial revolution in England. *The Journal of Interdisciplinary History*. 3(2), 225-259.
65. Crafts, NFR, Harley, CK. 1992. Output Growth and the British Industrial Revolution: A Restatement of the Crafts-Harley View. *The Economic History Review*. 45(4), 703-730.
66. Wrigley, EA. 2013. Energy and the English industrial revolution. *Philosophical Transactions of the Royal Society A: Mathematical, Physical and Engineering Sciences*. 371(1986), 20110568.
67. Crossley, D. 2004. "The rivers of the Sheffield area" in The Archaeology of Industrialization: Society of Post-Medieval Archaeology Monographs, D. Barker, D. Cranstone, Eds. London: Routledge.vol. 2, p. 333.
68. Reynolds, TS. 1983. *Stronger than a hundred men: a history of the vertical water wheel*. Baltimore: John Hopkins University Press. p. 453.
69. Bishop, P, Muñoz-Salinas, E. 2013. Tectonics, geomorphology and water mill location in Scotland, and the potential impacts of mill dam failure. *Applied Geography*. 42195-205.
70. Bishop, P. 2021. OS mapping of water mills. *Sheetlines*. 120.
71. Bowen, E, cartographer(s). 1729. *A New and accurate map of South Wales containing the counties of Pembroke, Glamorgan, Carmarthen, Brecknock, Cardigan and Radnor : wherein are exactly laid down and delineated from an actual survey and admeasurement all the towns, villages, churches, chaples, gentlemen's seats* [engraved map]. Scale: ca. 3 inches = 5 miles. London: Owen and Bowen. Available in National Library of Wales: https://viewer.library.wales/4997624#?xywh=1059%2C802%2C4725%2C2964
72. Jefferys, T, cartographer(s). 1751. *An accurate map of the County of Warwick Divided into its Hundreds, collected from the Best Materials Agreable to Sir William Dugdale's History, with various imrpovements made by several Persons residing in the County* [engraved map]. Scale: 77 mm = 6 Geometrical or Measur'd Miles. London: Publisher unknown. Available in McMaster University Library: http://digitalarchive.mcmaster.ca/islandora/object/macrepo%3A79688
73. Roque, J, cartographer(s). 1752. *To his royal highness George Prince of Wales &c &c, this actual survey of the County of Salop is most humbly inscrib'd by His most Dutiful and Most Obedient Servant, John Roque* [engraved map]. Scale: ca. 1 inch = 1 mile. London: Roque. Available in McMaster University Library: https://digitalarchive.mcmaster.ca/islandora/object/macrepo%3A81071

74. Roque, J, cartographer(s). 1752-1761. *A Topographical Map of the county of Berks* [engraved map]. Scale: ca. 15 cm = 3 miles. London: Roque. Available in National Library of France: https://gallica.bnf.fr/ark:/12148/btv1b530572216
75. Donn, B, cartographer(s). 1765. *A Map of the County of Devon, with the City & County of Exeter Delineated from an actual Survey by Benjamin Donn* [engraved map]. Scale: ca. 1:63,360. London: Jefferys. Available in Royal Geographical Society (with IBG) Wiley Digital Archives.
76. Jefferys, T, cartographer(s). 1765. *The County of Bedford Surveyed Anno MDCCLXV, and Engraved by Thomas Jefferys* [engraved map]. Scale: ca. 1:4,752. London: Faden. Available in the British Library: Not digitised
77. Armstrong, A, cartographer(s). 1768. *The County Palatine of Durham Survey'd By Capt. Armstrong and Engraved by Thomas Jefferys Geographer to His Majesty MDCCLXVIII* [engraved map]. Scale: 1 inch =1 mile. London: Jefferys. Available in National Library of Scotland: https://maps.nls.uk/joins/10430.html
78. Roque, J, Andrews, P, cartographer(s). 1768. *A Topographical Map of the County of Surrey In which is Expressed all the Roads, Lanes, Churches, Noblemen and Gentlemen's Seats &c. &c, of Principal Observations, By the Late John Roque, Topographer to His Majesty, Compleated and Engrav'd by Peter Andrews* [engraved map]. Scale: 2 inches = 1 mile. London: Roque. Available in Royal Geographical Society (with IBG) Wiley Digital Archives.
79. Armstrong, A, Armstrong, MJ, cartographer(s). 1769. *A map of the county of Northumberland with that part of the county of Durham that is north of the River Tyne, also the town of Berwick and its bounds ... Taken from an Actual Survey and laid down from a Scale of an Inch to a Mile* [engraved map]. Scale: 1 mile to an inch. London: Kitchin. Available in National Library of Scotland: https://maps.nls.uk/joins/10452.html
80. Jefferys, T, cartographer(s). 1769. *The County of Oxford, Surveyed Anno MDCCLXVI & VII; and Engraved by Thomas Jefferys, Geographer to His Majesty MDCCLXVIII* [engraved map]. Scale: ca. 1:63,360. London: Jefferys. Available in Yale University Library: https://collections.library.yale.edu/catalog/15309097
81. Jefferys, T, cartographer(s). 1770. *The County of Westmoreland, Surveyed Anno MDCCLXVIII and Engraved by Thomas Jefferys Geographer to His Majesty MDCCLXX* [engraved map]. Scale: 1 inch = 1 mile. London: Jefferys. Available in National Library of Scotland: https://maps.nls.uk/joins/10472.html
82. Jefferys, T, cartographer(s). 1770. *The County of Buckingham, Surveyed in MDCCLXVI, VII, and VIII and Engraved by Thomas Jefferys, Geographer to His Majesty, MDCCLXX* [engraved map]. Scale: 1 inch = 1 mile. London: Jefferys. Available in McMaster University Library: https://digitalarchive.mcmaster.ca/islandora/object/macrepo%3A79496
83. Stobie, M, cartographer(s). 1770. *A Map of Roxburghshire or Tiviotdale* [engraved map]. Scale: Not given. London: Publisher unknown. Available in National Library of Scotland, by permission of The Society of Writers to His Majesty's Signet: https://maps.nls.uk/joins/7431.html
84. Armstrong, A, Armstrong, MJ, Bell, A, cartographer(s). 1771. *Map of the County of Berwick Taken from an Actual, Survey and laid down from a Scale of an Inch to a Mile, By Capt. Armstrong and Son, & Engraved by A Bell 1771* [engraved map]. Scale: 1 inch

to the mile. Unknown: Bell. Available in National Library of Scotland: https://maps.nls.uk/joins/588.html
85. Jefferys, T, cartographer(s). 1771. *The County of York, survey'd in MDCCLXVII, VIII, IX and MDCCLXX; Engraved by Thomas Jefferys, Geographer to His Majesty, MDCCLXXI* [engraved map]. Scale: 1 inch = 1 mile. London: Jefferys. Available in McMaster University Library: http://digitalarchive.mcmaster.ca/islandora/object/macrepo%3A92720
86. Kirk, J, cartographer(s). 1772. *Book of Plans of the Parish of Loth, The Property of the Countess of Sutherland, containing threeteen different farms / surveyed and planed by John Kirk, 1772* [engraved map]. Scale: 1 inch = 10 Scots Chains. Sutherland Tutors. Available in National Library of Scotland, by permssion of the Countess of Sutherland: https://maps.nls.uk/estates/golspie-loth/graphic-index.html
87. Taylor, I, cartographer(s). 1772. *This Map of the County of Worcester, most Humbly Dedicated by their Obedient and most Humble Servant Isaac Taylor* [engraved map]. Scale: 1 inch = 1 mile. Available in McMaster University Library: http://digitalarchive.mcmaster.ca/islandora/object/macrepo%3A80895
88. Ainslie, J, cartographer(s). 1773. *A Map of Selkirk Shire or Ettrick Forest from a Survey taken in the Year 1772 By John Ainslie* [engraved map]. Scale: 1 mile = 8 furlongs. Edinburgh: Dickson and Veitch Available in National Library of Scotland: https://maps.nls.uk/view/74400327
89. Andrews, J, Dury, A, cartographer(s). 1773. *To Noblemen, Gentlemen, Clergy, Freeholders of the County of Wilts; This Map is Inscribed By their most Obedient and devoted Servants* [engraved map]. Scale: 2 inches = 1 mile. Available in McMaster University Library: http://digitalarchive.mcmaster.ca/islandora/object/macrepo%3A80926
90. Armstrong, A, Armstrong, MJ, cartographer(s). 1773. *To the nobility, gentry & clergy of the counties of Haddington, Edinburgh and Linlithgow this map of the Three Lothians is most humbly Inscribed By their much Obliged and most Obedient humble Survants Andrew & Mostyn Armstrong* [engraved map]. Scale: Scale not specified. Edinburgh: Kitchin. Available in National Library of Scotland: https://maps.nls.uk/joins/7206.html
91. Ainslie, J, cartographer(s). 1775. *The Counties of Fife and Kinross with the Rivers Forth and Tay Survey'd & Engraved By John Ainslie* [engraved map]. Scale: ca. 1 in = 1 mi. London: Faden and Jefferys. Available in National Library of Scotland: https://maps.nls.uk/joins/695.html
92. Armstrong, A, Armstrong, MJ, cartographer(s). 1775. *A New Map of Ayrshire comprehending Kyle, Cunningham & Carrick; The Scale, one Inch to a Mile; By Captain Armstrong and Son, and Engraved by S. Pyle MDCCLXXV* [engraved map]. Scale: 1 inch = 1 mile. Available in National Library of Scotland: https://maps.nls.uk/joins/797.html
93. Armstrong, MJ, cartographer(s). 1775. *To the Right Honourable William Douglas, Earl of March and Ruglen, Viscount Peebles, Lord Douglas of Neipath, Lyne, and Mannor, Knight of the most Antient Order of the Thistle, Lord Vice Admiral and one of the Sixteen Peers of Scotland and a Lord of his Majesty's Bed Chamber; This Map of the County of Peebles, or Tweedale, Is most humbly Inscribed By his Lordships much Obliged and most Obedient humble Servent Mostyn John Armstrong* [engraved map]. Scale: Scale not specified. Edinburgh: Pyle. Available in National Library of Scotland: https://maps.nls.uk/counties/rec/1020

94. Home, J, cartographer(s). 1775. *Survey of Assynt Plans No 1-16* [engraved map]. Scale: 1 inch = 20 Scots Chains. Edinburgh: Sutherland Tutors. Available in National Library of Scotland, by permission of the Countess of Sutherland: https://maps.nls.uk/estates/assynt/index.html
95. Yates, W, cartographer(s). 1775. *A Map of the County of Stafford from an actual survey begun in the year 1769 and finished in 1775; By William Yates; Engraved by John Chapman* [engraved map]. Scale: 1 inche = 1 mile. London: Chapman. Available in McMaster University Library: http://digitalarchive.mcmaster.ca/islandora/object/macrepo%3A92720
96. Garden, W, cartographer(s). 1776. *A map of Kincardinshire. Drawn from a survey taken anno MDCCLXXIV by William Garden* [engraved map]. Scale: Scale not specified. London: Begbie. Available in National Library of Scotland: https://maps.nls.uk/counties/rec/7202
97. Chapman, J, André, P, cartographer(s). 1777. *A Map of the County of Essex from an Actual Survey taken in MDCCLXXII : XXIII & MDCCLXXIV* [engraved map]. Scale: 2 inches = 1 mile Available in McMaster University Library: http://digitalarchive.mcmaster.ca/islandora/object/macrepo%3A79541
98. Ross, C, cartographer(s). 1777. *A Map of the Shire of Dumbarton Taken from an Actual Survey and laid down from a Scale of an Inch to a Mile by Charles Ross of Greenlaw* [engraved map]. Scale: 1 inch = 1 mile. Available in National Library of Scotland: https://maps.nls.uk/joins/595.html#gsc.tab=0
99. Taylor, I, cartographer(s). 1777. *To The Subscribers in General, and more Particularly To those Noblemen and Gentlemen who Honoured me with their Assistance in the Survey; This Map of the County of Gloucester Is Humbly Dedicated by their Obedient & most humble Servant Isaac Taylor* [engraved map]. Scale: 1 inch = 1 mile. Available in the British Library: Not digitised
100. Andrews, J, Dury, A, Herbert, W, cartographer(s). 1779. *A Topographical-Map of the County of Kent, in Twenty Five Sheets, on a Scale of two Inches to a Mile, from an Actual Survey; in which are Expressed all the Roads, Lanes, Churches, Towns, Villages, Noblemen and Gentlmens Seats, Roman Roads, Hills, Rivers, Woods, Cottages & every thing Remarkable in the County; Together with the Division of the Lathes & their Subdivision into Hundreds, By Jonathan Andrews, Andrew Dury & William Herbert* [engraved map]. Scale: 2 inches = 1 mile. London: Sayer and Bennett. Available in Royal Geographical Society (with IBG) Wiley Digital Archives.
101. Armstrong, A, cartographer(s). 1779. *Map of Lincoln-Shire, comprehending Lindsey, Kesteven & Holland, Surveyed in the Years 1776, 7, & 8, By Capt. Armstong; Engraved by Stephen Pyle MDCCLXXVIII* [engraved map]. Scale: ca. 1 inch = 1 mile. Pyle. Available in McMaster University Library: http://digitalarchive.mcmaster.ca/islandora/object/macrepo%3A92692
102. Prior, J, cartographer(s). 1779. *...this map of the county of Leicestershire from an actual survey, begun in the year 1775, and finished in the year 1777...* [engraved map]. Scale: 1 inch = 1 mile. London: Dawson and Gregory. Available in the British Library: Not digitised
103. Roque, J, cartographer(s). 1780. *A topographical Map of the County of Middlesex By John Roque* [engraved map]. Scale: ca. 1: 31,680. Available in Royal Geographical Society (with IBG) Wiley Digital Archives.

104. Ross, C, cartographer(s). 1780. *A Map of Stirling Shire from an Actual Survey by Charles Ross of Greenlaw* [engraved map]. Scale: Scale not specified. Lumsden. Available in National Library of Scotland: https://maps.nls.uk/counties/rec/6662
105. Armstrong, A, cartographer(s). 1781. *To the Nobility, Gentry, And all the Subscribers for the County of Rutland This Map, is most Humbly Inscribed By their most Obedient Humble Servent Captain Andrew Armstrong* [engraved map]. Scale: 1 inch = 1 mile. London: Laurie and Whittle. Available in Royal Geographical Society (with IBG) Wiley Digital Archives.
106. Ainslie, J, cartographer(s). 1782. *A Map of the County of Wigton or the Shire of Galloway Survey'd & Engrav'd by John Ainslie 1782* [engraved map]. Scale: 1 inch = 1 mile. Edinburgh: Ainslie. Available in National Library of Scotland: https://maps.nls.uk/counties/rec/685
107. Day, W, Masters, CH, cartographer(s). 1782. *County of Somerset Surveyed By Day and Masters MDCCLXXXII* [engraved map]. Scale: 1 inch = 1 mile. London: Faden. Available in McMaster University Library: https://digitalarchive.mcmaster.ca/islandora/object/macrepo%3A79589
108. Dury, A, Andrews, J, cartographer(s). 1782. *A Topographical Map of Hartford-Shire, From an Actual Survey; In which is Expressed all the Roads, Lanes, Churches, Noblemen and Gentlemen's Seats, and every Thing remarkable in the County: Together, with the Divsion of the Parishes* [engraved map]. Scale: 1 inch = 1 mile London: Faden. Available in Royal Geographical Society (with IBG) Wiley Digital Archives.
109. Donald, T, cartographer(s). 1783. *To the Most Honourable Charles Howard, Earl of Surrey, Lord Lieutenant and Custos Rotulorum of the West Riding in the County of York, and teh City of York, & County of the same, Deputy Earl Marshal of England, and One of the Lords Commisioners of His Majesty's Treasury, This Map of the County of Cumberland, Surveyed in 1770 & 1771, by Thomas Donald* [engraved map]. Scale: 1 inch = 1 mile. London: Hodskinson and Donald. Available in National Library of Scotland: https://maps.nls.uk/counties/rec/10481
110. Hodskinson, J, cartographer(s). 1783. *The County of Suffolk Surveyed By Joseph Hodskinson of Arundel Street, Strand* [engraved map]. Scale: 1 inch = 1 mile. London: Paden. Available in McMaster University Library: https://digitalarchive.mcmaster.ca/islandora/object/macrepo%3A79772
111. Stobie, J, cartographer(s). 1783. *The Counties of Perth and Clackmannan, Survey'd and Publish'd by James Stobie / Engraved by Thomas Conder, London, 1783* [engraved map]. Scale: Not given. London: Publisher Unknown. Available in National Library of Scotland: https://maps.nls.uk/joins/664.html
112. Martyn, T, cartographer(s). 1784. *A New and Accurate Map of the County of Cornwall from an Actual Survey* [engraved map]. London: Faden. Available in McMaster University Library: https://digitalarchive.mcmaster.ca/islandora/object/macrepo%3A92688
113. Snell, R, cartographer(s). 1785. *To The Nobility, Gentry, Clergy, and Others, This Map of the County of Monmouth, is Most Humbly, Inscribed By their Ever Dutyfull & Very Humble Servant Robert Snell* [engraved map]. Scale: Scale not specified. Bristol: Ames. Available in National Library of Wales: https://viewer.library.wales/1445598#?xywh=-2863%2C-529%2C17475%2C10963

114. Taylor, I, Faden, W, cartographer(s). 1786. *To My Worthy Subscribers in General but more particularly To those Noblemen Gentlemen &c. who honour'd me with their Assistance This New Map of the County of Hereford Engrav'd from the Original Drawing made from an Accurate Survey proved by Trigonometry Is most Humbly Dedicated by their Obedient Humble Servant, London, Isaac Taylor* [engraved map]. Scale: ca. 4cm = 5 miles. London: Taylor and Kitchin. Available in McMaster University Library: https://digitalarchive.mcmaster.ca/islandora/object/macrepo%3A81082
115. Yates, W, cartographer(s). 1786. *The County Palantine of Lancaster, Surveyed by William Yates, Engraved by Thomas Billinge* [engraved map]. Scale: ca. 1 inch = 1 mile. London. Available in National Library of Scotland: https://maps.nls.uk/joins/10434.html
116. Burdett, PP, cartographer(s). 1791. *To The Right Honourable The President, Vice-President And the rest of the Members of the Society, for the Encouragement of Arts, &c, This Survey of Derbyshire, Began in the Year 1762, and finished in the Year, Is Humbly Dedicated, By their much obliged Servant, P. P. Burdett* [engraved map]. Scale: 1 inch = 1 mile. London: Cary and Snowden. Available in National Library of Scotland: https://maps.nls.uk/joins/10424.html
117. Eyre, T, Jefferys, T, cartographer(s). 1791. *The County of Northampton as Surveyed and Planned by The late Mr. Thomas Eyre of Kettering, Revised By the Late Mr. Thomas Jefferys Geographer to the King, and Engraved by William Faden 1779* [engraved map]. Scale: ca. 1 inch = 1 mile. London: Faden. Available in McMaster University Library: https://digitalarchive.mcmaster.ca/islandora/object/macrepo%3A79480
118. Milne, T, cartographer(s). 1791. *Hampshire, or the County of Southampton, including the Isle of Wight. Surveyed by Thomas Milne in the Years 1788, 89, & 90, executed and Published at the private expence of the Proprietor W. Faden, Geographer to His Majesty* [engraved map]. London: Faden. Available in Royal Geographical Society (with IBG) Wiley Digital Archives.
119. Yates, W, cartographer(s). 1793. *A Map of Warwickshire drawn from an Actual Survey taken in the Years 1787-1788-1789 by William Yates & Sons For John Sharp* [engraved map]. Scale: 1 inch = 1 mile. Publisher unknown. Available in McMaster University Library: https://digitalarchive.mcmaster.ca/islandora/object/macrepo%3A81079
120. Ainslie, J, cartographer(s). 1794. *Map of the County of Forfar or Shire of Angus From an Actual Survey by Join Ainslie Landsurveyer* [engraved map]. Scale: ca. 2 inches = 1 mile. Edinburgh: Ainslie. Available in National Library of Scotland: https://maps.nls.uk/joins/577.html
121. Burdett, PP, cartographer(s). 1794. *By Permission, To His Royal Highness, George, Prince of Wales, Duke of Corwall & Rothsay, and Earl of Chester, &c. &c. This Survey of the County Palantine of Chester, is most humbly Dedicate, by His Royal Highness's most Devoted Servant, P.P. Burdett* [engraved map]. Scale: ca. 1 inch = 1 mile. London: Faden. Available in Royal Geographical Society (with IBG) Wiley Digital Archives. https://maps.nls.uk/view/220113193
122. Chapman, J, cartographer(s). 1795. *Nottinghamshire Survey'd in 1774 by John Chapman* [engraved map]. Scale: 1 inch = 1 mile. London: Faden. Available in National Library of Scotland: https://maps.nls.uk/joins/10466.html
123. Evans, J, cartographer(s). 1795. *To Sir Watkin Williams-Wynn Barrister LLD, Member of Parliament, Lord Lieutenant and Custos Retulorum of the Counties of Denbigh and Merioneth, Colonel of the Antient British Fencible Cavalry, Steward of the Hundreds of*

Bromfield and Yal And Vice President of the Welsh Charity School, This Map of the Six Counties of North-Wales Is most humbly Inscribed by his obedient humble Servant John Evans [engraved map]. Scale: 3/4 inch = 1 mile. Llwyn-y-groes [?]: Evans. Available in National Library of Wales: https://www.library.wales/discover-learn/digital-exhibitions/maps/maps-of-wales/evans-map-of-north-wales
124. Gream, T, Gardner, W, Yeakell, T, cartographer(s). 1795. *A Topographical Map of the County of Sussex, divided into Rapes, Deanries, and Hundreds, Planned from an actual Survey by a Scale of one Inch to a Statute Mile, begun by William Gardner and the late Thomas Yeakell, Completed by Thomas Gream* [engraved map]. Scale: 1 inch = 1 mile. London: Faden. Available in McMaster University Library: https://digitalarchive.mcmaster.ca/islandora/object/macrepo%3A80922
125. Taylor, I, cartographer(s). 1795. *The County of Dorset, Survey and Engraved by Isaac Taylor...revised and corrected* [engraved map]. Scale: 1 inch = 1 mile. London: Faden. Available in Royal Geographical Society (with IBG) Wiley Digital Archives. https://digitalarchive.mcmaster.ca/islandora/object/macrepo%3A79504
126. Ainslie, J, cartographer(s). 1797. *Stewartry of Kirkcudbright by John Ainslie Landserveyor Edinburgh* [engraved map]. Scale: ca. 1 inch = 1 mile. Edinburgh: Ainslie. Available in National Library of Scotland: https://maps.nls.uk/joins/637.html
127. Donald, T, Milne, T, cartographer(s). 1797. *A Topographical Map of the County of Norfolk Surveyed and Measured in the YEars 1790, 91, 92, 93 and 94, By Thomas Donald, Thomas Milne, and Assistants, Planned from a Scale of one Inch to a Statue Mile, The Whole Executed and Published at the expence of the Proprietor William Faden, Geographer to His Majesty and His Royal Highness the Prince of Wales* [engraved map]. Scale: 1 inch = 1 mile. London: Faden. Available in McMaster University Library: https://digitalarchive.mcmaster.ca/islandora/object/macrepo%3A79583
128. Yates, G, cartographer(s). 1799. *A Map of the County of Glamorgan; From an Actual Survey, made By George Yates of Liverpool. On which Are Delineated teh Course of the Rivers, and Navigable Canals; with The Roads, Parks, Gentlemens Seats, Castles, Woods, &c. &c.* [engraved map]. London: Cary. Available in National Library of Wales: https://viewer.library.wales/4997601
129. Ainslie, J, cartographer(s). 1800. *Map of the County of Renfrew Surveyed by John Ainslie in 1796* [engraved map]. Scale: ca. 2 inches = 1 mile. London: Faden. Available in National Library of Scotland: https://maps.nls.uk/joins/669.html
130. Day, W, Masters, CH, cartographer(s). 1800. *County of Somerset Surveyed By Day and Masters MDCCLXXXII* [engraved map]. Scale: 1 inch = 1 mile. London: Faden. Available in Royal Geographical Society (with IBG) Wiley Digital Archives.
131. Langlands, G, Son, cartographer(s). 1801. *This Map of Argyllshire Taken from Actual Survey is most humbly dedicated To His Grace John Duke of Argyll &c.&c.& By His Graces most Obliged, & most humble Servants, George Landlands & Son* [engraved map]. Campbeltown: Langlands. Available in National Library of Scotland, by permission of The Society of Writers to His Majesty's Signet: https://maps.nls.uk/joins/7382.html
132. Forrest, W, cartographer(s). 1802. *Map of Haddington Shire surveyed by William Forrest 1799* [engraved map]. Scale: ca. 1 inch =1 mile. Edinburgh: Forrest. Available in National Library of Scotland: https://maps.nls.uk/joins/629.html

133. Singer, J, cartographer(s). 1803. *A New Map of Cardiganshire, from an Actual Survey, made By Joseph Singer, Land Surveyor: on which are described its Hundreds, Market-Towns, Parishes, Hamlets, Roads, Rivers, Gentlemen's Seats, Heaths Mines &c.* [engraved map]. London: Cary. Available in National Library of Wales: https://viewer.library.wales/1445593#?xywh=-4297%2C3373%2C22997%2C14427
134. Crawford, W, cartographer(s). 1804. *Map of Dumfries-shire From an Actual Survey by William Crawford* [engraved map]. Scale: 1 inch = 1 mile. Edinburgh: Crawford. Available in National Library of Scotland: https://maps.nls.uk/joins/597.html
135. Jefferys, T, cartographer(s). 1804. *The County of Huntingdon, Surveyed Anno MDCCLXVI, and Engraved by Thomas Jefferys Geographer to His Majesty, Anno MDCCLXVIII* [engraved map]. London: Faden. Available in Royal Geographical Society (with IBG) Wiley Digital Archives.
136. Forrest, W, cartographer(s). 1816. *The County of Lanark From Actual Survey, William Forrest* [engraved map]. Scale: ca 2 inches = 1 mile. Edinburgh: Forrest. Available in National Library of Scotland: https://maps.nls.uk/joins/646.html
137. Price, H, cartographer(s). 1817. *A New Map of the County of Hereford with part of the Adjacent Counties from an Actual Survey by Henry Price; and founded on the Basis of the Trigonometrical Survey of the Kingdom by Colonel Mudge under the Authority of His Majesty's Honorable Board of Ordnance* [engraved map]. London: Price. Available in Royal Geographical Society (with IBG) Wiley Digital Archives.
138. Greenwood, C, cartographer(s). 1818. *Map of the County Palatine of Lancaster, from Actual Survey; made in the Year 1818 By C Greenwood* [engraved map]. Scale: 1 inch = 1 mile. London: Fowler & Greenwood. Available in National Library of Scotland: https://maps.nls.uk/joins/10442.html
139. Greenwood, C, cartographer(s). 1819. *Map of the County of Middlesex, from Actual Survey made in The Years 1818 & 1819 By C Greenwood* [engraved map]. Scale: 2 inches = 1 mile. London: Princle and Greenwood. Available in Royal Geographical Society (with IBG) Wiley Digital Archives.
140. Greenwood, C, cartographer(s). 1820. *Map of the County Palatine of Durham From Actual Survey made in the Years 1818 & 1819 By C Greenwood* [engraved map]. Scale: 1 inch = 1 mile. London: Greenwood, Pringle & Co. Available in National Library of Scotland: https://maps.nls.uk/counties/rec/10482
141. Greenwood, C, cartographer(s). 1820. *Map of the County of Stafford from Actual Survey made in the Years 1818 & 1819 By C Greenwood* [engraved map]. Scale: 1 inch = 1 mile. London: Fowler, Greenwood & Co. Available in Royal Geographical Society (with IBG) Wiley Digital Archives.
142. Thomson, J, Johnson, W, cartographer(s). 1820. *Linlithgow-Shire* [engraved map], in John Thomson's 1832 Atlas of Scotland. Scale: ca. 1.5 inches = 1 mile. Edinburgh: Thomson & Co. Available in National Library of Scotland: https://maps.nls.uk/atlas/thomson/451.html
143. Thomson, J, Johnson, W, cartographer(s). 1820. *Sutherland Shire* [engraved map], in John Thomson's 1832 Atlas of Scotland. Scale: ca. 1.5 inches = 1 mile. Edinburgh: Thomson & Co. Available in National Library of Scotland: https://maps.nls.uk/atlas/thomson/505.html
144. Thomson, J, Johnson, W, Blackadder, J, Chapman, J, cartographer(s). 1820. *Skye Island &c* [engraved map], in John Thomson's 1832 Atlas of Scotland. Scale: ca. 1.5 inches = 1

mile. Edinburgh: Thomson & Co. Available in National Library of Scotland: https://maps.nls.uk/view/74400149
145. Thomson, J, Johnson, W, Grassom, J, cartographer(s). 1820. *Stirling-Shire* [engraved map], in John Thomson's 1832 Atlas of Scotland. Scale: ca. 1.5 inches = 1 mile. Edinburgh: Thomson & Co. Available in National Library of Scotland: https://maps.nls.uk/atlas/thomson/453.html
146. Ainslie, J, cartographer(s). 1821. *Ainslie's Map of the Southern Part of Scotland* [engraved map]. Edinburgh: Macredie Skelly & Co. Available in National Library of Scotland: https://maps.nls.uk/joins/649.html
147. Greenwood, C, cartographer(s). 1821. *Map of the County of Kent, from an Actual Survey made in the Years 1819 & 1820, By C Greenwood* [engraved map]. Scale: 1 inch = 1 mile. London: Pringle Jr. Available in Royal Geographical Survey (with IBG) Wiley Digital Archives.
148. Thomson, J, Johnson, W, Blackadder, J, cartographer(s). 1821. *Berwick-Shire* [engraved map], in John Thomson's 1832 Atlas of Scotland. Scale: ca. 1.5 inches = 1 mile. Edinburgh: Thomson & Co. Available in National Library of Scotland: https://maps.nls.uk/atlas/thomson/455.html
149. Thomson, J, Johnson, W, Little, J, MacKenzie, C, cartographer(s). 1821. *Peebles-Shire* [engraved map], in John Thomson's 1832 Atlas of Scotland. Scale: ca. 1.5 inches = 1 mile. Edinbrugh: Thomson & Co. Available in National Library of Scotland: https://maps.nls.uk/counties/rec/7280
150. Bryant, A, cartographer(s). 1822. *Map of the County of Hertford, From an Actual Survey By A Bryant In the Years 1820 and 1821* [engraved map]. Scale: 1.5 inches = 1 mile. London: Bryant. Available in Royal Geographical Society (with IBG) Wiley Digital Archives.
151. Greenwood, C, Greenwood, J, cartographer(s). 1822. *Map of the County of Somerset, from an Actual Survey made in the Years 1820 & 1821, By C & J Greenwood* [engraved map]. Scale: 1 inch = 1 mile. London: Pringle Jr. Available in Royal Geographical Society (with IBG) Wiley Digital Archives.
152. Robertson, J, cartographer(s). 1822. *To General, the Most Noble, the Marquis of Huntly, Baron Gordon, G.C.B., &c.&c and Lord Lieutentant of Aberdeenshire, This Topographical and Military Map of the Counties of Aberdeen, Banff, and Kincardine; with Parts of those of Forfar, Perth, Inverness, and Moray; exhibiting a true Picture thereof, constructed and delineated, from actual surveys, on Trigonometrical, perspective, and optical principles; being the second of this kind yet published, my Map of Jamaica having been the first; Is Most respectifully inscribed, By His Lordship's Most Obliged and most faithful humble servant, James Robertson, A.M. F.R.S.* [engraved map]. Scale: 1 inch = 1 mile. London: Roberston. Available in National Library of Scotland: https://maps.nls.uk/joins/570.html
153. Thomson, J, Crawford, W, Son, cartographer(s). 1822. *Roxburgh Shire* [engraved map], in John Thomson's 1832 Atlas of Scotland. Scale: ca. 1.5 inch = 1 mile. Edinburgh: Thomson & Co. Available in National Library of Scotland: https://maps.nls.uk/atlas/thomson/479.html
154. Thomson, J, Johnson, W, Sinclair, J, cartographer(s). 1822. *Caithness-Shire* [engraved map], in John Thomson's Atlas of Scotland. Scale: ca. 1.5 inches = 1 mile. Edinburgh:

Thomson & Co. Available in National Library of Scotland: https://maps.nls.uk/atlas/thomson/485.html
155. Thomson, J *et al.*, cartographer(s). 1822-1823. *Western Isles* [engraved map], in John Thomson's 1832 Atlas of Scotland. Scale: ca. 1.5 inches = 1mile. Edinburgh: Thomson & Co. Available in National Library of Scotland: https://maps.nls.uk/atlas/thomson/494.html
156. Greenwood, C, Greenwood, J, cartographer(s). 1823. *Map of the County of Cumberland, From an Actual Survey Made in The Years 1821 & 1822, By C and J Greenwood, Most Respectfully Dedicated to the Nobility, Clergy & Gentry of the Country By The Proprietors* [engraved map]. Scale: 1 inch = 1 mile. Pringle Jr. Available in National Library of Scotland: https://maps.nls.uk/joins/10421.html
157. Thomson, J, Johnson, W, Bauchope, R, cartographer(s). 1823. *Buteshire* [engraved map], in John Thomson's 1832 Atlas of Scotland. Scale: ca. 1.5 inches = 1 mile. Edinburgh: Thomson & Co. Available in National Library of Scotland: https://maps.nls.uk/atlas/thomson/502.html
158. Bryant, A, cartographer(s). 1824. *Map of the County of Oxford, From actual Survey, By A Bryant In the Year 1823, Inscribed by Permission to the Right Honorable the Earl of Macclesfield, Lord Lieutenant and to the Nobility, Clergy, and Gengry of the County* [engraved map]. Scale: 1.5 inches = 1 mile. London: Bryant. Available in Yale University Library
https://collections.library.yale.edu/catalog/15309102
159. Bryant, A, cartographer(s). 1824. *Map of the County of Surrey From Actual Survey by A Bryant in the Years 1822 & 1823* [engraved map]. Scale: 1.5 inches = 1 mile. London: Bryant. Available in Royal Geographical Society (with IBG) Wiley Digital Archives.
160. Greenwood, C, Greenwood, J, cartographer(s). 1824. *Map of the County of Westmorland, From an Actual Survey made in The Years 1822 & 1823 By C & J Greenwood, Most Respectfully Dedicated to the Nobility, Clergy & Gentry of the County* [engraved map]. Scale: 1 inch = 1 mile. London: Greenwood, Pringle & Co. Available in National Library of Scotland: https://maps.nls.uk/counties/rec/10486
161. Greenwood, C, Greenwood, J, cartographer(s). 1824. *Map of the County of Gloucester, From an Actual Survey Made in The Year 1823, By C and J Greenwood, Most Respectfully; Dedicated to the Nobility, Clergy & Gentry of the County, by the Proprietors Greenwood, Pringle & Co* [engraved map]. Scale: 1 inch = 1 mile. London: Greenwood, Pringle & Co. Available in Royal Geographical Society (with IBG) Wiley Digital Archives.
162. Thomson, J, Johnson, W, cartographer(s). 1824. *Argyllshire* [engraved map], in John Thomson's 1832 Atlas of Scotland. Edinburgh: Thomson & Co. Available in National Library of Scotland: https://maps.nls.uk/view/74400147
163. Thomson, J, Johnson, W, Mitchell, T, Kinghorne, A, Kinghorne, J, cartographer(s). 1824. *Selkirk Shire* [engraved map], in John Thomson's 1832 Atlas of Scotland. Scale: ca. 1.5 inches = 1 mile. Edinburgh: Thomson & Co. Available in National Library of Scotland: https://maps.nls.uk/atlas/thomson/512.html
164. Bryant, A, cartographer(s). 1825. *Map of the County of Buckingham From Actual Survey, By A Bryant, In the Year 1824* [engraved map]. Scale: 1.5 inches = 1 mile. London: Bryant. Available in The British Library (not digitized).:

165. Greenwood, C, Fowler, W, Sharp, T, cartographer(s). 1825. *Map of the County of Haddington made on the basis of the Trigonometrical Survey of Scotland Surveyed in the Years 1824 and 1825 and Published by the Proprietors Thomas Sharp, C Greenwood & William Fowler* [engraved map]. Scale: 1 inch = 1 mile. London: Sharp, Greenwood & Fowler. Available in National Library of Scotland: https://maps.nls.uk/joins/633.html
166. Greenwood, C, Greenwood, J, cartographer(s). 1825. *Map of the County of Essex, From an Actual Survey made in the Year 1824, by C & J Greenwood, most respectfully Dedicated to the Nobility, Clergy and Gentry of the County by the Proprietors, Greenwood, Pringle & Co* [engraved map]. Scale: 1 inch = 1 mile. London: Greenwood, Pringle & Co. Available in Royal Geographical Society (with IBG) Wiley Digital Archives.
167. Thomson, J, Johnson, W, Blackadder, W, cartographer(s). 1825. *Angus Shire* [engraved map], in John Thomson's 1832 Atlas of Scotland. Scale: ca. 1.5 inches = 1 mile. Edinburgh: Thomson & Co. Available in National Library of Scotland: https://maps.nls.uk/atlas/thomson/516.html
168. Bryant, A, cartographer(s). 1826. *Map of the County of Suffolk, From Actual Survey, By A Bryant, In the Years 1824 and 1825* [engraved map]. Scale: 1.25 inches = 1 mile. London: Bryant. Available in Royal Geographical Society (with IBG) Wiley Digital Archives.
169. Bryant, A, cartographer(s). 1826. *Map of the County of Bedford from Actual Survey by A Bryant, In the Years 1825 and 1826* [engraved map]. Scale: 1.5 inches = 1 mile. London: Bryant. Available in Royal Geographical Society (with IBG) Wiley Digital Archives.
170. Greenwood, C, Greenwood, J, cartographer(s). 1826. *Map of the County of Nottingham, From an Actual Survey made in The Years 1824 & 1825* [engraved map]. Scale: 1 inch = 1 mile. London: Greenwood, Pringle & Co. Available in National Library of Scotland: https://maps.nls.uk/joins/10470.html
171. Greenwood, C, Greenwood, J, cartographer(s). 1826. *Map of the County of Dorset from an Actual Survey made in the Years 1825 & 1826, By C & J Greenwood* [engraved map]. Scale: 1 inch = 1 mile. London: Greenwood, Pringle & Co. Available in Royal Geographical Society (with IBG) Wiley Digital Archives.
172. Thomson, J, Craig, J, cartographer(s). 1826. *Ross and Cromarty Shires* [engraved map], in John Thomson's 1832 Atlas of Scotland. Scale: ca. 1.5 inches = 1 mile. Edinburgh: Thomson. Available in National Library of Scotland: https://maps.nls.uk/counties/rec/7316
173. Thomson, J, Johnson, W, cartographer(s). 1826. *County of Wigton or Shire of Galloway* [engraved map], in John Thomson's 1832 Atlas of Scotland. Scale: ca. 1.5 inches = 1 mile. Edinburgh: Thomson & Co. Available in National Library of Scotland: https://maps.nls.uk/view/74400164
174. Thomson, J, Johnson, W, Lamb, J, cartographer(s). 1826. *Renfrew-Shire* [engraved map], in John Thomson's 1832 Atlas of Scotland. Scale: ca. 1.5 inches = 1 mile. Edinburgh: Edinburgh. Available in National Library of Scotland: https://maps.nls.uk/view/74400165
175. Thomson, J, Ross, AI, cartographer(s). 1826. *Aberdeen & Banff-Shires* [engraved map], in John Thomson's 1832 Atlas of Scotland. Scale: ca. 1.5 inch = 1 mile. Edinburgh: Thomson & Co. Available in National Library of Scotland: https://maps.nls.uk/atlas/thomson/523.html
176. Bryant, A, cartographer(s). 1827. *Map of the County of Northampton from Actual Survey By A Bryant, in the Years 1824, 1825, 1826, Respectfully Dedicated To the Nobility,*

Clergy, and Gentry of the County [engraved map]. Scale: 1.5 inches = 1 mile. London: Bryant. Available in Yale University Library: https://collections.britishart.yale.edu/catalog/orbis:584335

177. Campbell, JC, cartographer(s). 1827. *Map of the County of Pembroke Made from an Actual Survey in the Year 1826 By J C Campbell* [engraved map]. Scale: 1 inch = 1 mile. London: Campbell & Walley. Available in National Library of Wales: https://viewer.library.wales/4997595#?xywh=1302%2C4161%2C4034%2C1391
178. Greenwood, C, Greenwood, J, cartographer(s). 1827. *Map of the County of Devon From an Actual Survey Made in the Years 1825 & 1826, By C & J Greenwood, Most Respectfully Dedicated to the Nobility, Clergy and Gentry of the County By the Proprietors Greenwood, Pringle & Co* [engraved map]. Scale: 1 inch = 1 mile. London: Greenwood, Pringle & Co. Available in Royal Geographical Society (with IBG) Wiley Digital Archives.
179. Greenwood, C, Greenwood, J, cartographer(s). 1827. *Map of the County of Cornwall, From an Actual Survey made in the Years 1826 & 1827, By C & J Greenwood* [engraved map]. Scale: 1 inch = 1 mile. London: Greenwood & Co. Available in Royal Geographical Society (with IBG) Wiley Digital Archives.
180. Thomson, J, Johnson, W, cartographer(s). 1827. *Perthshire* [engraved map], in John Thomson's 1832 Atlas of Scotland. Scale: ca. 1.5 inches = 1 mile. Edinburgh: Thomson & Co. Available in National Library of Scotland: https://maps.nls.uk/joins/7238.html
181. Bryant, A, cartographer(s). 1828. *Map of the County of Lincoln, From Actual Survey made in the Years 1825-26 & 27, By A Bryant, Respectfully Dedicated To The Nobility, Clergy & Gentry, of County* [engraved map]. Scale: 1 inch = 1 mile. London: Bryant. Available in National Library of Scotland: https://maps.nls.uk/joins/10448.html
182. Greenwood, C, Bingley, J, Teesdale, H, cartographer(s). 1828. *To the Nobility, Gentry, & Clergy, of Yorkshire, this Map of the County constructed from a Survey commended in the Year 1817, & corrected in the Years 1827 & 1828, Is respectfully dedicated by the Proprietors* [engraved map]. Scale: 1 inch = 1 1/4 miles. London: Teesdale & Co. Available in National Library of Scotland: https://maps.nls.uk/joins/10476.html
183. Greenwood, C, Fowler, W, Sharp, T, cartographer(s). 1828. *Map of the County of Edinburgh, made on the basis of the Trigonometrical Survey of Scotland, Surveyed in the Years 1827 and 1828, And Published by the Proprietors Thomas Sharp, Christopher Greenwood, and William Fowler* [engraved map]. Scale: 1 inch = 1 mile. London: Sharp, Greenwood, & Fowler. Available in National Library of Scotland: https://maps.nls.uk/joins/612.html
184. Greenwood, C, Greenwood, J, cartographer(s). 1828. *Map of the South East Circuit of the Principality of Wales comprising the Counties of Glamorgan, Brecon & Radnor, From an Actual Survey made in the Years 1826 &1827, By C & J Greenwood* [engraved map]. Scale: 3/4 inch = 1 mile. London: Greenwood & Co. Available in National Library of Wales: https://viewer.library.wales/4997599#?xywh=1592%2C2701%2C7847%2C2706
185. Sharp, T, Greenwood, C, Fowler, W, cartographer(s). 1828. *Map of the Counties of Fife and Kinross, made on the basis of the Trigonometrical Survey of Scotland, Surveyed in the Years 1826 and 1827, And Published by the Proprietors, Thomas Sharp, C Greenwood, and William Fowler* [engraved map]. Scale: 1 inch = 1 mile. London: Sharp, Greenwood & Fowler. Available in National Library of Scotland, by permission of The Society of Writers to His Majesty's Signet: https://maps.nls.uk/counties/rec/7417

186. Thomson, J, Crawford, W, Son, cartographer(s). 1828. *Dumfriesshire* [engraved map], in John Thomson's 1832 Atlas of Scotland. Scale: ca. 1.5 miles = 1 mile. Edinburgh: Thomson & Co. Available in National Library of Scotland: https://maps.nls.uk/atlas/thomson/555.html
187. Thomson, J, Johnson, W, cartographer(s). 1828. *Ayrshire* [engraved map], in John Thomson's 1832 Atlas of Scotland. Scale: ca. 1.5 inches = 1 mile. Edinburgh: Thomson. Available in National Library of Scotland: https://maps.nls.uk/joins/7166.html
188. Greenwood, C, Greenwood, J, cartographer(s). 1829. *Map of the County of Wilts, from an Actual Survey made in the Years 1819 & 1820, By C & J Greenwood...corrected to the present period* [engraved map]. Scale: ca. 1.5 inch = 1 mile. London: Greenwood & Co. Available in Yale University Library: https://collections.library.yale.edu/catalog/15309127
189. Greenwood, C, Greenwood, J, cartographer(s). 1829. *Map of the County of Sussex From an actual Survey made in the years 1823 and 1824, By C & J Greenwood...corrected to the present period* [engraved map]. Scale: 1 inch = 1 mile. London: Greenwood & Co. Available in Yale University Library: https://collections.library.yale.edu/catalog/15309071
190. Greenwood, C, Greenwood, J, cartographer(s). 1829. *Map of the County of Southampton. from an Actual Survey made in the Years 1825 & 1826 By C & J Greenwood...corrected to the present period* [engraved map]. Scale: 1.5 inches = 1 mile. London: Greenwood & Co. Available in David Rumsey Map Center, Stanford Libraries:
191. Greenwood, C, Greenwood, J, cartographer(s). 1829. *Map of the County of Berks, from an Actual Survey made in the Years 1822 & 1823, By C & J Greenwood, Published by the Proprietors Greenwood & Co... Corrected to the present period* [engraved map]. Scale: 1 inch = 1 mile. London: Greenwood & Co. Available in Yale University Library: https://collections.library.yale.edu/catalog/15309121
192. Home, J, Kirk, J, cartographer(s). 1829. *Volume of 13 plans of Golspie and environs, 1767-1829* [engraved map]. Scale: Scale not specified. Available in National Library of Scotland, by permssion of the Countess of Sutherland: https://maps.nls.uk/estates/golspie-loth/graphic-index.html
193. Greenwood, C, Greenwood, J, cartographer(s). 1830. *Map of the County of Derby, from an Actual Survey made in the Years 1824 & 1825, by C & J Greenwood...corrected to the present period* [engraved map]. Scale: 1 inch = 1 mile. London: Greenwood & Co. Available in David Rumsey Map Center, Stanford Libraries:
194. Greenwood, C, Greenwood, J, cartographer(s). 1830. *Map of the County of Warwick from an Actual Survey made in the year 1821 By C& J Greenwood...corrected to the present period* [engraved map]. Scale: 1 inch = 1 mile. London: Greenwood & Co. Available in Yale University Library: https://collections.library.yale.edu/catalog/15309132
195. Greenwood, C, Greenwood, J, cartographer(s). 1830. *Map of the County of Leicester, from an Actual Survey made in the Year 1825, by C & J Greenwood, Published by the Proprietors Greenwood & Co...Corrected to the present period* [engraved map]. Scale: 1 inch = 1 mile. London: Greenwood & Co. Available in David Rumsey Map Center, Stanford Libraries: https://www.davidrumsey.com/luna/servlet/detail/RUMSEY~8~1~319348~90088265:Map-o

196. Greenwood, C, Greenwood, J, cartographer(s). 1830. *Map of the County of Salop. From an Actual Survey made in the Years 1826 & 1827. By C & J Greenwood...corrected to the present period* [engraved map]. Scale: 1 inch = 1 mile. London: Greenwood & Co. Available in Yale University Library: https://collections.library.yale.edu/catalog/15309197
197. Greenwood, C, Greenwood, J, cartographer(s). 1830. *Map of the County of Huntingdon, from an Actual Survey, made in the Year 1829, by C & J Greenwood, Published by the Proprietors Greenwood & Co...corrected to the present period* [engraved map]. Scale: ca. 1.5 inches = 1 mile. London: Greenwood & Co. Available in Yale University Library: https://collections.library.yale.edu/catalog/15350025
198. Greenwood, C, Greenwood, J, cartographer(s). 1830. *Map of the County of Stafford From an Actual Survey Made in the Years 1818 & 1819, By C & J Greenwood; Published by the Proprietors Greenwood & Co...corrected to the present period* [engraved map]. Scale: 1 inch = 1 mile. London: Greenwood & Co. Available in Yale University Library: https://collections.library.yale.edu/catalog/15350009
199. Greenwood, C, Greenwood, J, cartographer(s). 1830. *Map of the County of Worcester from an Actual Survey made in the Years 1820 & 1821 By C & J Greenwood, Published by the Proprietors Green & Co...Corrected to the Present Period* [engraved map]. Scale: ca. 1.5 inches = 1 mile. London: Greenwood & Co. Available in Yale University Library: https://collections.library.yale.edu/catalog/15350015
200. Greenwood, C, Greenwood, J, cartographer(s). 1830. *Map of the County of Monmouth, From an Actual Survey made in the Years 1829 & 1830, By C & H Greenwood* [engraved map]. Scale: 1 inch = 1 mile. London: Greenwood & Co. Available in National Library of Wales: https://viewer.library.wales/4997597#?xywh=8023%2C1345%2C4541%2C1566
201. Thomson, J, Johnson, W, cartographer(s). 1830. *Inverness Shire* [engraved map], in John Thomson's 1832 Atlas of Scotland. Scale: ca. 1.5 inches = 1 mile. Edinburgh: Thomson & Co. Available in National Library of Scotland: https://maps.nls.uk/atlas/thomson/487.html
202. Thomson, J, Johnson, W, cartographer(s). 1830. *Nairn and Elgin* [engraved map], in John Thomson's 1832 Atlas of Scotland. Scale: ca. 1.5 inch = 1 mile. Edinburgh: Thomson & Co. Available in National Library of Scotland: https://maps.nls.uk/atlas/thomson/566.html
203. Bryant, A, cartographer(s). 1831. *Map of the County Palatine of Chester From an Actual Survey made in the Years 1829, 1830 & 1831* [engraved map]. Scale: 1 1/4 inches = 1 mile. London: Bryant. Available in National Library of Scotland: https://maps.nls.uk/joins/10417.html
204. Greenwood, C, Greenwood, J, cartographer(s). 1831. *Map of the County of Northumberland from an Actual Survey in the Years 1827 & 1828 By C & J Greenwood...corrected to the present period* [engraved map]. Scale: 1 inch = 1 mile. London: Greenwood & Co. Available in David Rumsey Map Center, Stanford Libraries: https://www.davidrumsey.com/luna/servlet/detail/RUMSEY~8~1~319354~90088427:Map-o
205. Burnett, G, Scott, W, cartographer(s). 1833. *Map of the County of Sutherland; Made on the basis of the Trigonometrical Survey of Scotland, in the years 1831-1832 By Gregory Burnett and William Scott, Surveyors to His Grace the Duke of Sutherland, K.G.*

[engraved map]. Available in National Library of Scotland: https://maps.nls.uk/view/216588931
206. Greenwood, C, Greenwood, J, cartographer(s). 1834. *Map of the County of Cambridge, from an Actual Survey made in the Years 1832 & 1833, By C & J Greenwood, Published by the Proprietors Greenwood & Co...Corrected to the present period* [engraved map]. Scale: ca. 1 inch = 1 mile. London: Greenwood & Co. Available in Yale University Library: https://collections.library.yale.edu/catalog/15349986
207. Greenwood, C, Greenwood, J, cartographer(s). 1834. *Map of the County of Norfolk, From an Actual Survey made in the Years 1831 and 1832 By C & J Greenwood, Published by the Proprietors Greenwood & Co...Corrected to the Present Period* [engraved map]. Scale: ca. 1 inch = 1 mile. London: Greenwood & Co. Available in Yale University Library: https://collections.library.yale.edu/catalog/15309111
208. King, W, cartographer(s). 1836. *A map of a tract of country surrounding Belvoir Castle; Including extensive districts of the counties of Leicester, Lincoln & Nottingham; and the whole of the county of Rutland...from a survey taken in the Years 1804, 5, & 6...* [engraved map]. Scale: ca. 1 inch = 1 mile. London: Faden. Available in The British Library (not digitized).:
209. Sanderson, G, cartographer(s). 1836. *Map of the County of Derby, From a Careful Survey made in the Years 1834 and 1835, Is with the greatest respect Inscribed to John Coke Esq of Debdale, One of His Majesty's Justice of Peace, for the County* [engraved map]. Scale: 1 inch = 1 mile. London: Sanderson. Available in the British Library: Not digitised
210. Harley, JB. 1972. *Maps for the local historian: a guide to the British sources*. London: The Standing Conference for Local History / National Council for Social Service. p. 86.
211. Lee, RJ. 1955. *English County Maps: The Identification, Cataloguing, and Physical Care of a Collection*. London: Library Association. p. 32.
212. Bishop, P. 2022. OS 25-inch mapping of threshing mills in Scotland. *Sheetlines*. 1237-25.
213. Kanefsky, J, Townley, J. 2023. Keep that wheel a-turning: why and how the hybrid of steam pumping engine and water wheel combinations was appropriate technology in changing times. in *Second International Early Engines Conference*, eds S. Grudgings, P. Stephens. Dudley, UK: IEEC in collab. with the International Stationary Steam Engine Society, pp 49-62.
214. Evans, O. 1795. *The Young Mill-Wright and Miller's Guide*. Philadelphia: Evans, Oliver. p. 352.
215. Evans, O. 1826. *The Young Steam Engineer's Guide*. Philadelphia: H.C. Carey & I. Lea. p. 139.
216. Evans, O. 1805. *The Abortion of the Young Steam Engineer's Guide*. Philadelphia: Fry & Kammerer. p. 139.
217. Anonymous. 1812. MILL. in *The Cyclopaedia; or Universal Dictionary of Arts, Sciences and Literature*, ed A. Rees. London: London: Longman, Hurst, Rees, Orme & Brown.
218. Farey Jr., J, Others. 1818. WATER. in *The Cyclopædia; or, Universal dictionary of arts, sciences, and literature*, ed A. Rees. London: Longman, Hurst, Rees, Orme & Brown, p 807.
219. Farey Jr., J, Others. 1816. STEAM. in *The Cyclopaedia; or Universal Dictionary of Arts, Sciences and Literature*, ed A. Rees. London: Longman, Hurst, Rees, Orme & Brown.

220. Farey Jr., J. 1827. *A treatise on the steam engine, historical, practical, and descriptive*. London: Longman, Rees, Orme, Brown & Green. p. 1048.
221. Butterworth, E. 1856. *Historical Sketches of Oldham... With an appendix containing the history of the town to the present time*.John Hirst.
222. Hills, RL. 1970. *Power in the Industrial Revolution*. Manchester: Manchester University Press. p. 274.
223. Aikin, JD. 1795. *A description of the country from thirty to forty miles round Manchester*. London: Stockdale.
224. Association, MLECoTB. 1962. *Manchester and its region: A survey prepared for the British Association* C. F. Carter, Ed. Manchester: Manchester University Press for the British Association.
225. Downward, S, Skinner, K. 2005. Working rivers: the geomorphological legacy of English freshwater mills. *Area*. 37(2), 138–147.
226. Walter, RC, Merritts, DJ. 2008. Natural streams and the legacy of water-powered mills. *Science*. 319(5861), 299-304.
227. Bishop, P, Jansen, J. 2005. The geomorphological setting of some of Scotland's east coast freshwater mills: a comment on Downward and Skinner (2005) 'Working rivers: the geomorphological legacy...'. *Area*. 37(4), 443–445.
228. Chapman, SD. 1970. Fixed capital formation in the British cotton industry, 1770–1815. *The Economic History Review*. 23(2), 235-253.
229. Unwin, G. 1924. *Samuel Oldknow and the Arkwrights: The industrial revolution at Stockport and Marple*.The University Press.
230. Tupling, GH. 1927. *The economic history of Rossendale*. Manchester: Manchester University Press. p. 274.
231. Ashmore, O. 1969. *The Industrial Archaeology of Lancashire*.Newton Abbot: David & Charles. p. 352.
232. Pelham, RA. 1963. The water-power crisis in Birmingham in the eighteenth century. *University of Birmingham Historical Journal*. IX64.
233. Jenkins, DT, Ponting, KG. 1982. *British wool textile industry, 1770-1914*. London: Heinemann International. p. 388.
234. Rodgers, H. 1960. The Lancashire cotton industry in 1840. *Transactions and Papers (Institute of British Geographers)*. (28), 135-153.
235. Cunningham, W. 1913. "The Growth of English Industry and Commerce" in Modern Times. Cambridge: Cambridge University Press.vol. 2, p. 771.
236. Redford, A. 1931. *The Economic History of England, 1760-1860*. London: Longmans, Green. p. 221.
237. Landes, DS. 1969. *The Unbound Prometheus: Technical Change and Industrial Development in Western Europe from 1750 to Present* ed. 1. Cambridge: Cambridge University Press. p. 366.
238. Wrigley, EA. 1988. *Continuity, chance and change*. Cambridge: Cambridge University Press. p. 146.
239. Wilkinson, RG. 1973. *Poverty and Progress: An Ecological Model of Economic Development*. London: Routledge. p. 254.
240. Pomeranz, K. 2000. *The Great Divergence: China, Europe, and the Making of the Modern World Economy*. Princeton: Princeton University Press. p. 382.

241. Wrigley, EA. 1987. *People, cities and wealth: The transformation of traditional society*.Blackwell Oxford. p. 348.
242. Mokyr, J, Ed., *The British Industrial Revolution: an economic perspective.* Westview, Oxford, 1999, p 368.
243. Warde, P. 2007. *Energy consumption in England and Wales, 1560-2004*. Naples: Consiglio della Ricerche. p. 138.
